# Supplementary figures and images for: Mangrove species classification using a proposed ensemble U-Net model and Planet satellite imagery: A case study in Ngoc Hien district, Ca Mau province, Vietnam (part 2 of 2)
Source: PLoS One. 2025 Aug 6;20(8):e0327315. doi: 10.1371/journal.pone.0327315 (PMC12327635; doi:10.1371/journal.pone.0327315)

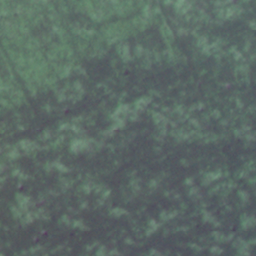

Supplement: S15 File — (ZIP) [file pone.0327315.s015.zip › INPUT_DONE/train_images/train/IMAGE_CaMau.tifpatch_128.tif]

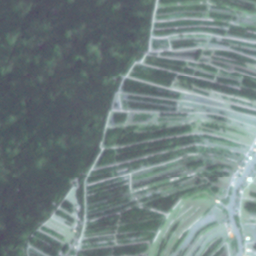

Supplement: S15 File — (ZIP) [file pone.0327315.s015.zip › INPUT_DONE/train_images/train/IMAGE_CaMau.tifpatch_1310.tif]

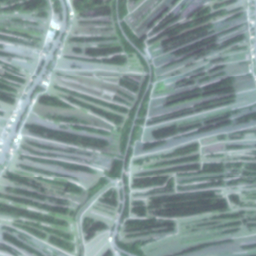

Supplement: S15 File — (ZIP) [file pone.0327315.s015.zip › INPUT_DONE/train_images/train/IMAGE_CaMau.tifpatch_1311.tif]

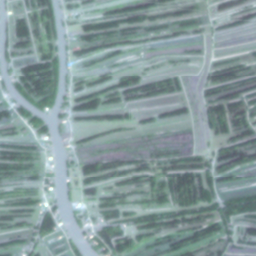

Supplement: S15 File — (ZIP) [file pone.0327315.s015.zip › INPUT_DONE/train_images/train/IMAGE_CaMau.tifpatch_1312.tif]

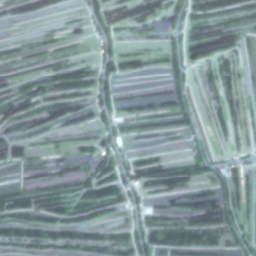

Supplement: S15 File — (ZIP) [file pone.0327315.s015.zip › INPUT_DONE/train_images/train/IMAGE_CaMau.tifpatch_1313.tif]

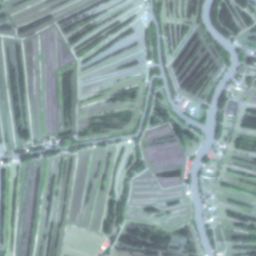

Supplement: S15 File — (ZIP) [file pone.0327315.s015.zip › INPUT_DONE/train_images/train/IMAGE_CaMau.tifpatch_1314.tif]

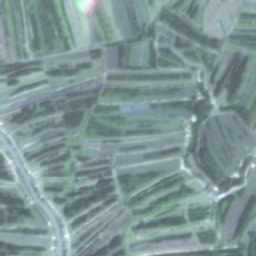

Supplement: S15 File — (ZIP) [file pone.0327315.s015.zip › INPUT_DONE/train_images/train/IMAGE_CaMau.tifpatch_1316.tif]

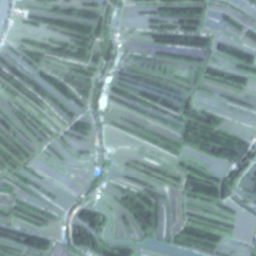

Supplement: S15 File — (ZIP) [file pone.0327315.s015.zip › INPUT_DONE/train_images/train/IMAGE_CaMau.tifpatch_1318.tif]

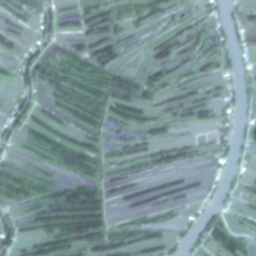

Supplement: S15 File — (ZIP) [file pone.0327315.s015.zip › INPUT_DONE/train_images/train/IMAGE_CaMau.tifpatch_1319.tif]

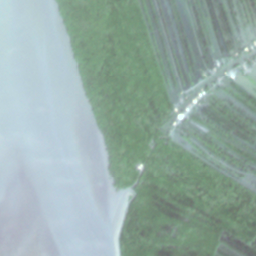

Supplement: S15 File — (ZIP) [file pone.0327315.s015.zip › INPUT_DONE/train_images/train/IMAGE_CaMau.tifpatch_132.tif]

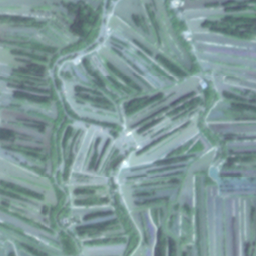

Supplement: S15 File — (ZIP) [file pone.0327315.s015.zip › INPUT_DONE/train_images/train/IMAGE_CaMau.tifpatch_1320.tif]

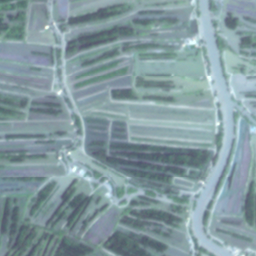

Supplement: S15 File — (ZIP) [file pone.0327315.s015.zip › INPUT_DONE/train_images/train/IMAGE_CaMau.tifpatch_1321.tif]

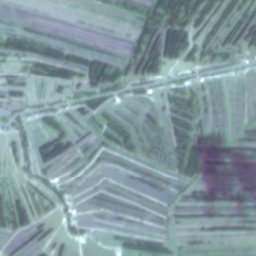

Supplement: S15 File — (ZIP) [file pone.0327315.s015.zip › INPUT_DONE/train_images/train/IMAGE_CaMau.tifpatch_1322.tif]

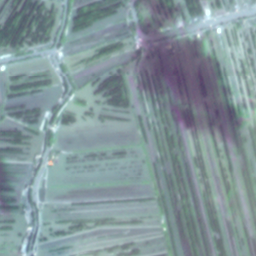

Supplement: S15 File — (ZIP) [file pone.0327315.s015.zip › INPUT_DONE/train_images/train/IMAGE_CaMau.tifpatch_1323.tif]

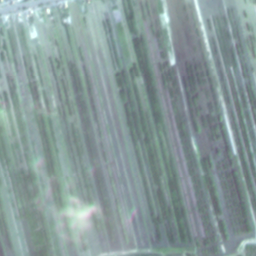

Supplement: S15 File — (ZIP) [file pone.0327315.s015.zip › INPUT_DONE/train_images/train/IMAGE_CaMau.tifpatch_1324.tif]

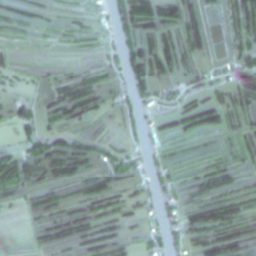

Supplement: S15 File — (ZIP) [file pone.0327315.s015.zip › INPUT_DONE/train_images/train/IMAGE_CaMau.tifpatch_1326.tif]

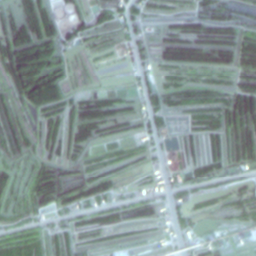

Supplement: S15 File — (ZIP) [file pone.0327315.s015.zip › INPUT_DONE/train_images/train/IMAGE_CaMau.tifpatch_1328.tif]

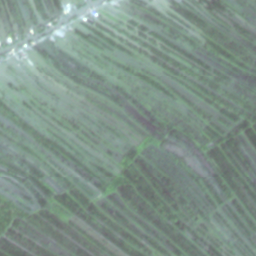

Supplement: S15 File — (ZIP) [file pone.0327315.s015.zip › INPUT_DONE/train_images/train/IMAGE_CaMau.tifpatch_133.tif]

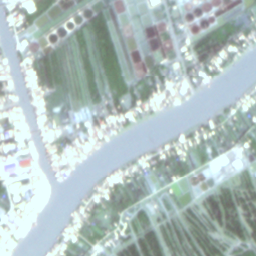

Supplement: S15 File — (ZIP) [file pone.0327315.s015.zip › INPUT_DONE/train_images/train/IMAGE_CaMau.tifpatch_1330.tif]

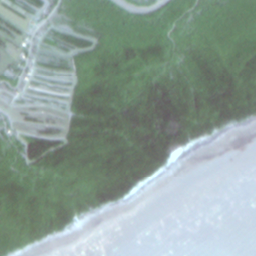

Supplement: S15 File — (ZIP) [file pone.0327315.s015.zip › INPUT_DONE/train_images/train/IMAGE_CaMau.tifpatch_1332.tif]

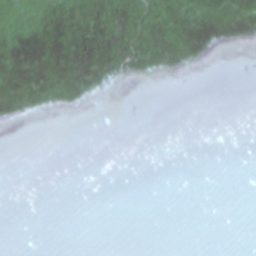

Supplement: S15 File — (ZIP) [file pone.0327315.s015.zip › INPUT_DONE/train_images/train/IMAGE_CaMau.tifpatch_1333.tif]

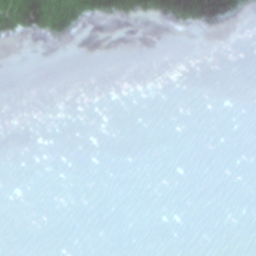

Supplement: S15 File — (ZIP) [file pone.0327315.s015.zip › INPUT_DONE/train_images/train/IMAGE_CaMau.tifpatch_1334.tif]

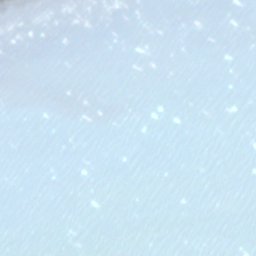

Supplement: S15 File — (ZIP) [file pone.0327315.s015.zip › INPUT_DONE/train_images/train/IMAGE_CaMau.tifpatch_1335.tif]

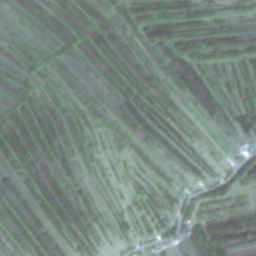

Supplement: S15 File — (ZIP) [file pone.0327315.s015.zip › INPUT_DONE/train_images/train/IMAGE_CaMau.tifpatch_134.tif]

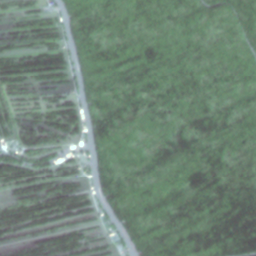

Supplement: S15 File — (ZIP) [file pone.0327315.s015.zip › INPUT_DONE/train_images/train/IMAGE_CaMau.tifpatch_135.tif]

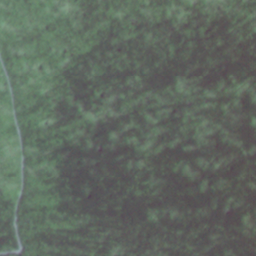

Supplement: S15 File — (ZIP) [file pone.0327315.s015.zip › INPUT_DONE/train_images/train/IMAGE_CaMau.tifpatch_136.tif]

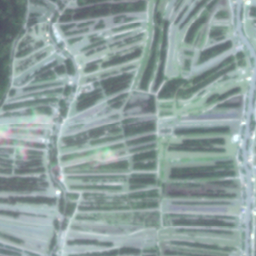

Supplement: S15 File — (ZIP) [file pone.0327315.s015.zip › INPUT_DONE/train_images/train/IMAGE_CaMau.tifpatch_1410.tif]

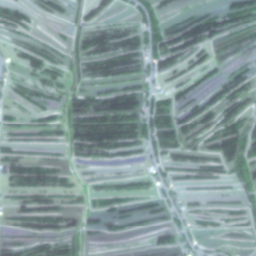

Supplement: S15 File — (ZIP) [file pone.0327315.s015.zip › INPUT_DONE/train_images/train/IMAGE_CaMau.tifpatch_1411.tif]

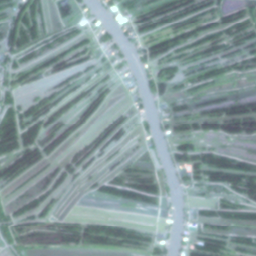

Supplement: S15 File — (ZIP) [file pone.0327315.s015.zip › INPUT_DONE/train_images/train/IMAGE_CaMau.tifpatch_1412.tif]

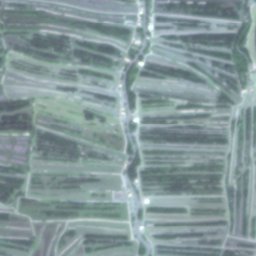

Supplement: S15 File — (ZIP) [file pone.0327315.s015.zip › INPUT_DONE/train_images/train/IMAGE_CaMau.tifpatch_1413.tif]

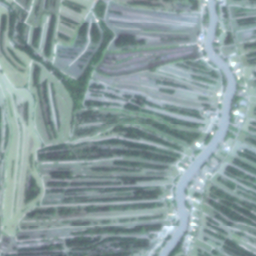

Supplement: S15 File — (ZIP) [file pone.0327315.s015.zip › INPUT_DONE/train_images/train/IMAGE_CaMau.tifpatch_1414.tif]

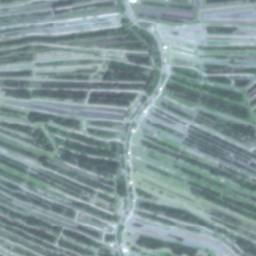

Supplement: S15 File — (ZIP) [file pone.0327315.s015.zip › INPUT_DONE/train_images/train/IMAGE_CaMau.tifpatch_1415.tif]

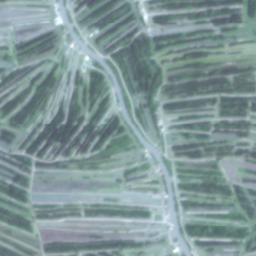

Supplement: S15 File — (ZIP) [file pone.0327315.s015.zip › INPUT_DONE/train_images/train/IMAGE_CaMau.tifpatch_1416.tif]

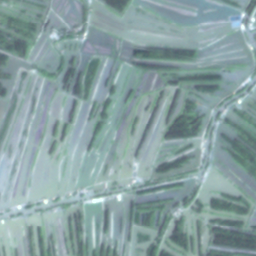

Supplement: S15 File — (ZIP) [file pone.0327315.s015.zip › INPUT_DONE/train_images/train/IMAGE_CaMau.tifpatch_1418.tif]

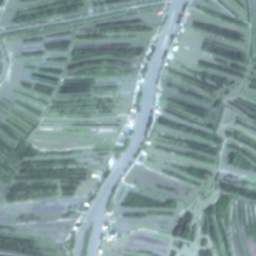

Supplement: S15 File — (ZIP) [file pone.0327315.s015.zip › INPUT_DONE/train_images/train/IMAGE_CaMau.tifpatch_1419.tif]

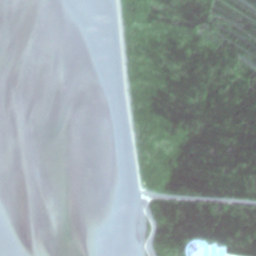

Supplement: S15 File — (ZIP) [file pone.0327315.s015.zip › INPUT_DONE/train_images/train/IMAGE_CaMau.tifpatch_142.tif]

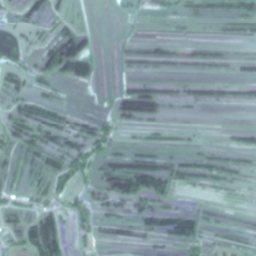

Supplement: S15 File — (ZIP) [file pone.0327315.s015.zip › INPUT_DONE/train_images/train/IMAGE_CaMau.tifpatch_1422.tif]

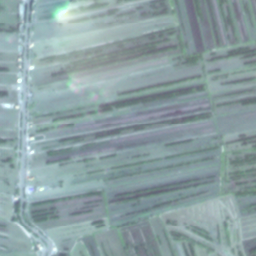

Supplement: S15 File — (ZIP) [file pone.0327315.s015.zip › INPUT_DONE/train_images/train/IMAGE_CaMau.tifpatch_1423.tif]

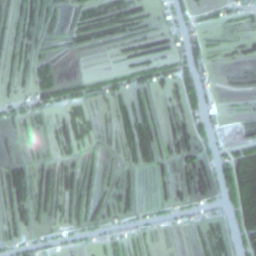

Supplement: S15 File — (ZIP) [file pone.0327315.s015.zip › INPUT_DONE/train_images/train/IMAGE_CaMau.tifpatch_1425.tif]

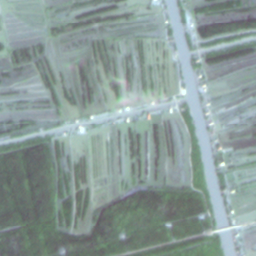

Supplement: S15 File — (ZIP) [file pone.0327315.s015.zip › INPUT_DONE/train_images/train/IMAGE_CaMau.tifpatch_1426.tif]

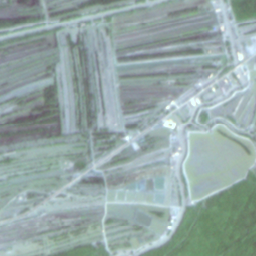

Supplement: S15 File — (ZIP) [file pone.0327315.s015.zip › INPUT_DONE/train_images/train/IMAGE_CaMau.tifpatch_1427.tif]

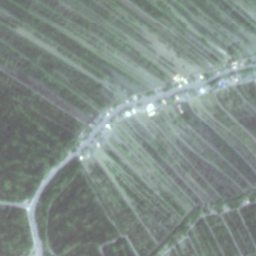

Supplement: S15 File — (ZIP) [file pone.0327315.s015.zip › INPUT_DONE/train_images/train/IMAGE_CaMau.tifpatch_143.tif]

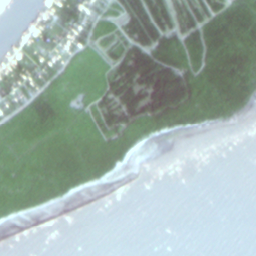

Supplement: S15 File — (ZIP) [file pone.0327315.s015.zip › INPUT_DONE/train_images/train/IMAGE_CaMau.tifpatch_1430.tif]

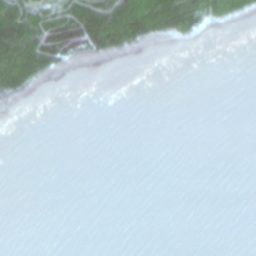

Supplement: S15 File — (ZIP) [file pone.0327315.s015.zip › INPUT_DONE/train_images/train/IMAGE_CaMau.tifpatch_1431.tif]

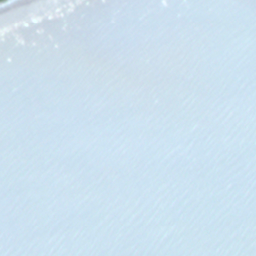

Supplement: S15 File — (ZIP) [file pone.0327315.s015.zip › INPUT_DONE/train_images/train/IMAGE_CaMau.tifpatch_1432.tif]

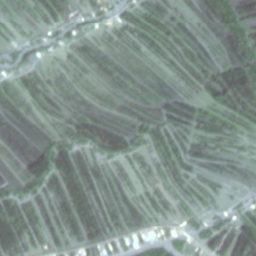

Supplement: S15 File — (ZIP) [file pone.0327315.s015.zip › INPUT_DONE/train_images/train/IMAGE_CaMau.tifpatch_144.tif]

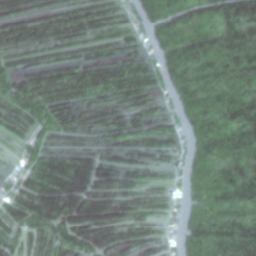

Supplement: S15 File — (ZIP) [file pone.0327315.s015.zip › INPUT_DONE/train_images/train/IMAGE_CaMau.tifpatch_145.tif]

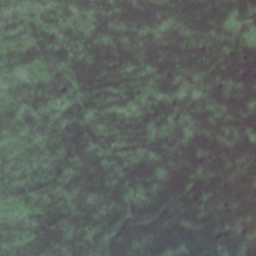

Supplement: S15 File — (ZIP) [file pone.0327315.s015.zip › INPUT_DONE/train_images/train/IMAGE_CaMau.tifpatch_146.tif]

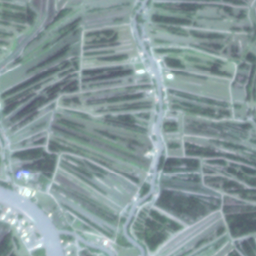

Supplement: S15 File — (ZIP) [file pone.0327315.s015.zip › INPUT_DONE/train_images/train/IMAGE_CaMau.tifpatch_1513.tif]

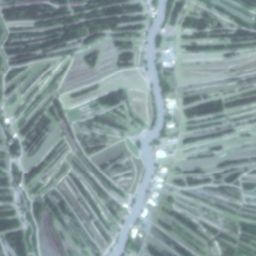

Supplement: S15 File — (ZIP) [file pone.0327315.s015.zip › INPUT_DONE/train_images/train/IMAGE_CaMau.tifpatch_1514.tif]

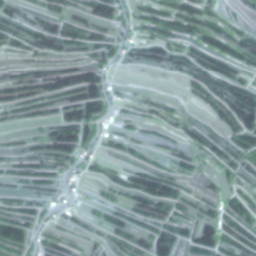

Supplement: S15 File — (ZIP) [file pone.0327315.s015.zip › INPUT_DONE/train_images/train/IMAGE_CaMau.tifpatch_1515.tif]

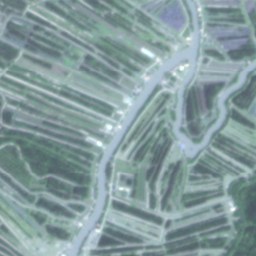

Supplement: S15 File — (ZIP) [file pone.0327315.s015.zip › INPUT_DONE/train_images/train/IMAGE_CaMau.tifpatch_1516.tif]

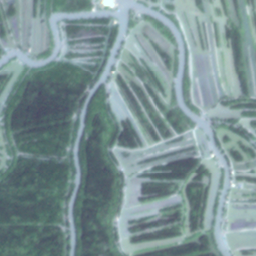

Supplement: S15 File — (ZIP) [file pone.0327315.s015.zip › INPUT_DONE/train_images/train/IMAGE_CaMau.tifpatch_1517.tif]

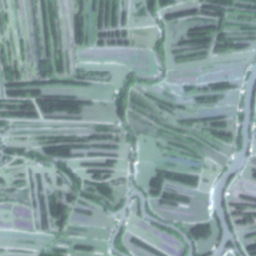

Supplement: S15 File — (ZIP) [file pone.0327315.s015.zip › INPUT_DONE/train_images/train/IMAGE_CaMau.tifpatch_1518.tif]

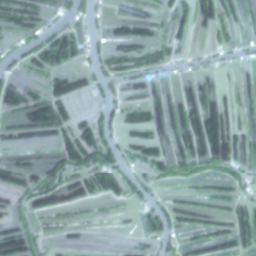

Supplement: S15 File — (ZIP) [file pone.0327315.s015.zip › INPUT_DONE/train_images/train/IMAGE_CaMau.tifpatch_1519.tif]

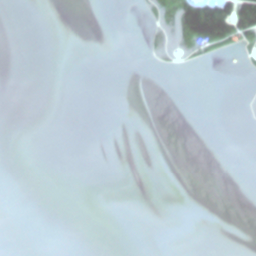

Supplement: S15 File — (ZIP) [file pone.0327315.s015.zip › INPUT_DONE/train_images/train/IMAGE_CaMau.tifpatch_152.tif]

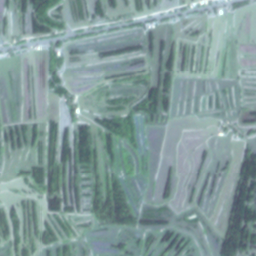

Supplement: S15 File — (ZIP) [file pone.0327315.s015.zip › INPUT_DONE/train_images/train/IMAGE_CaMau.tifpatch_1520.tif]

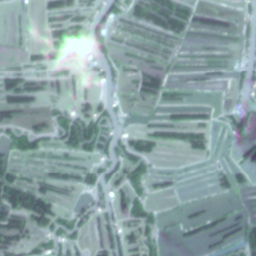

Supplement: S15 File — (ZIP) [file pone.0327315.s015.zip › INPUT_DONE/train_images/train/IMAGE_CaMau.tifpatch_1521.tif]

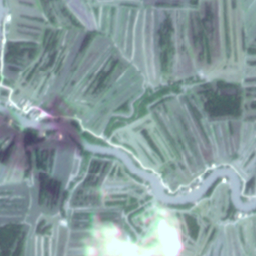

Supplement: S15 File — (ZIP) [file pone.0327315.s015.zip › INPUT_DONE/train_images/train/IMAGE_CaMau.tifpatch_1522.tif]

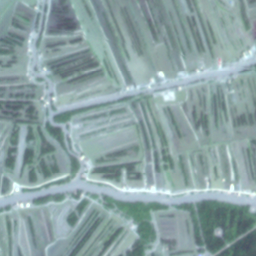

Supplement: S15 File — (ZIP) [file pone.0327315.s015.zip › INPUT_DONE/train_images/train/IMAGE_CaMau.tifpatch_1523.tif]

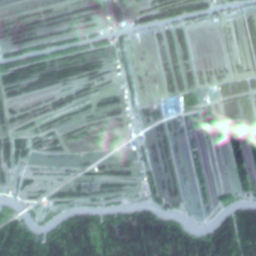

Supplement: S15 File — (ZIP) [file pone.0327315.s015.zip › INPUT_DONE/train_images/train/IMAGE_CaMau.tifpatch_1524.tif]

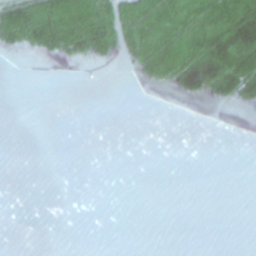

Supplement: S15 File — (ZIP) [file pone.0327315.s015.zip › INPUT_DONE/train_images/train/IMAGE_CaMau.tifpatch_1527.tif]

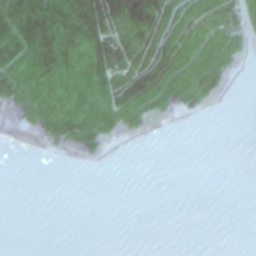

Supplement: S15 File — (ZIP) [file pone.0327315.s015.zip › INPUT_DONE/train_images/train/IMAGE_CaMau.tifpatch_1528.tif]

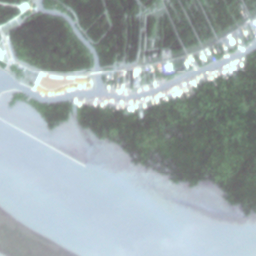

Supplement: S15 File — (ZIP) [file pone.0327315.s015.zip › INPUT_DONE/train_images/train/IMAGE_CaMau.tifpatch_153.tif]

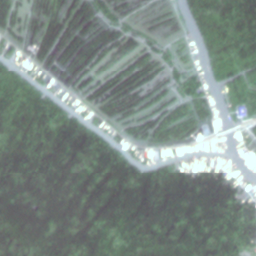

Supplement: S15 File — (ZIP) [file pone.0327315.s015.zip › INPUT_DONE/train_images/train/IMAGE_CaMau.tifpatch_155.tif]

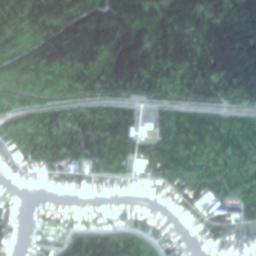

Supplement: S15 File — (ZIP) [file pone.0327315.s015.zip › INPUT_DONE/train_images/train/IMAGE_CaMau.tifpatch_156.tif]

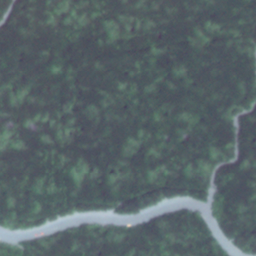

Supplement: S15 File — (ZIP) [file pone.0327315.s015.zip › INPUT_DONE/train_images/train/IMAGE_CaMau.tifpatch_158.tif]

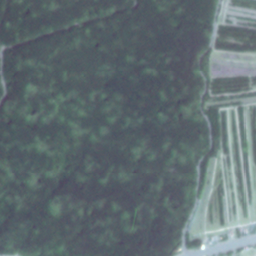

Supplement: S15 File — (ZIP) [file pone.0327315.s015.zip › INPUT_DONE/train_images/train/IMAGE_CaMau.tifpatch_159.tif]

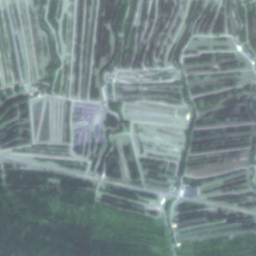

Supplement: S15 File — (ZIP) [file pone.0327315.s015.zip › INPUT_DONE/train_images/train/IMAGE_CaMau.tifpatch_1610.tif]

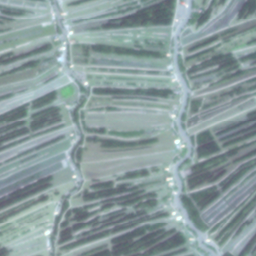

Supplement: S15 File — (ZIP) [file pone.0327315.s015.zip › INPUT_DONE/train_images/train/IMAGE_CaMau.tifpatch_1612.tif]

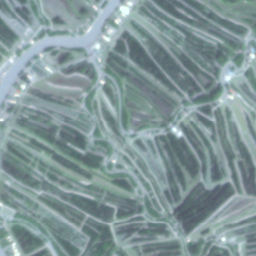

Supplement: S15 File — (ZIP) [file pone.0327315.s015.zip › INPUT_DONE/train_images/train/IMAGE_CaMau.tifpatch_1614.tif]

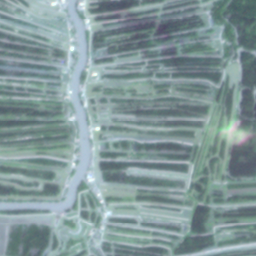

Supplement: S15 File — (ZIP) [file pone.0327315.s015.zip › INPUT_DONE/train_images/train/IMAGE_CaMau.tifpatch_1616.tif]

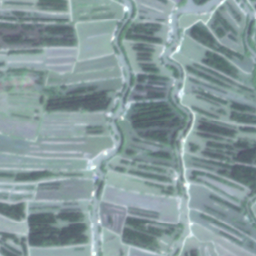

Supplement: S15 File — (ZIP) [file pone.0327315.s015.zip › INPUT_DONE/train_images/train/IMAGE_CaMau.tifpatch_1618.tif]

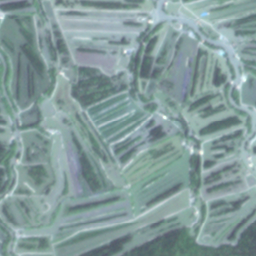

Supplement: S15 File — (ZIP) [file pone.0327315.s015.zip › INPUT_DONE/train_images/train/IMAGE_CaMau.tifpatch_1619.tif]

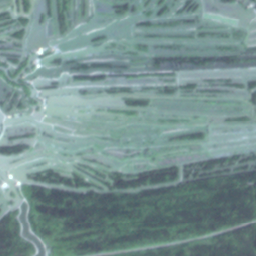

Supplement: S15 File — (ZIP) [file pone.0327315.s015.zip › INPUT_DONE/train_images/train/IMAGE_CaMau.tifpatch_1620.tif]

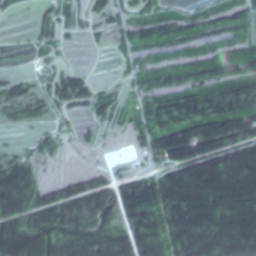

Supplement: S15 File — (ZIP) [file pone.0327315.s015.zip › INPUT_DONE/train_images/train/IMAGE_CaMau.tifpatch_1621.tif]

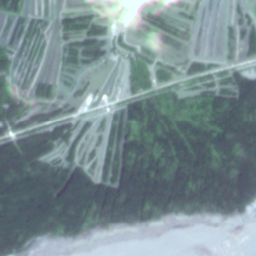

Supplement: S15 File — (ZIP) [file pone.0327315.s015.zip › INPUT_DONE/train_images/train/IMAGE_CaMau.tifpatch_1622.tif]

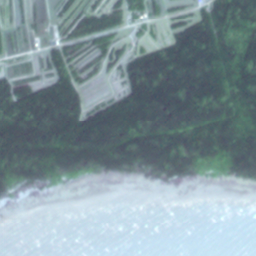

Supplement: S15 File — (ZIP) [file pone.0327315.s015.zip › INPUT_DONE/train_images/train/IMAGE_CaMau.tifpatch_1623.tif]

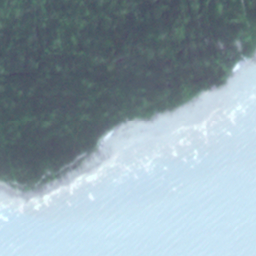

Supplement: S15 File — (ZIP) [file pone.0327315.s015.zip › INPUT_DONE/train_images/train/IMAGE_CaMau.tifpatch_1624.tif]

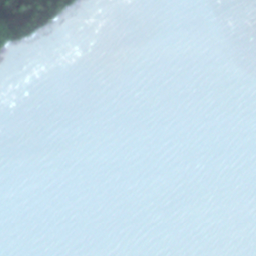

Supplement: S15 File — (ZIP) [file pone.0327315.s015.zip › INPUT_DONE/train_images/train/IMAGE_CaMau.tifpatch_1625.tif]

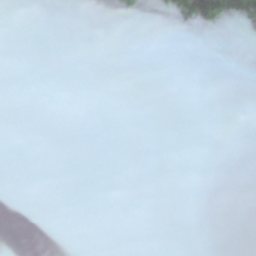

Supplement: S15 File — (ZIP) [file pone.0327315.s015.zip › INPUT_DONE/train_images/train/IMAGE_CaMau.tifpatch_164.tif]

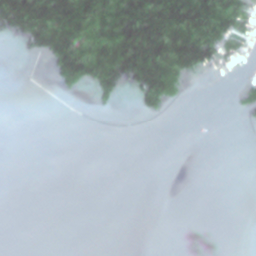

Supplement: S15 File — (ZIP) [file pone.0327315.s015.zip › INPUT_DONE/train_images/train/IMAGE_CaMau.tifpatch_165.tif]

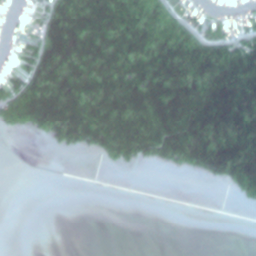

Supplement: S15 File — (ZIP) [file pone.0327315.s015.zip › INPUT_DONE/train_images/train/IMAGE_CaMau.tifpatch_166.tif]

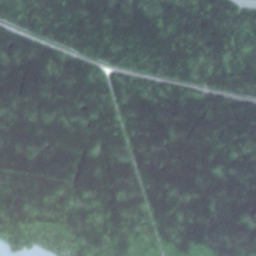

Supplement: S15 File — (ZIP) [file pone.0327315.s015.zip › INPUT_DONE/train_images/train/IMAGE_CaMau.tifpatch_168.tif]

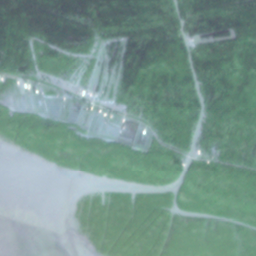

Supplement: S15 File — (ZIP) [file pone.0327315.s015.zip › INPUT_DONE/train_images/train/IMAGE_CaMau.tifpatch_1710.tif]

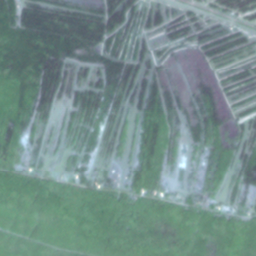

Supplement: S15 File — (ZIP) [file pone.0327315.s015.zip › INPUT_DONE/train_images/train/IMAGE_CaMau.tifpatch_1711.tif]

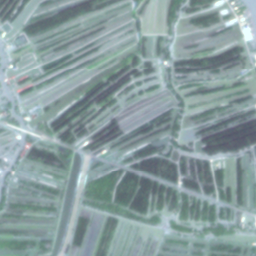

Supplement: S15 File — (ZIP) [file pone.0327315.s015.zip › INPUT_DONE/train_images/train/IMAGE_CaMau.tifpatch_1713.tif]

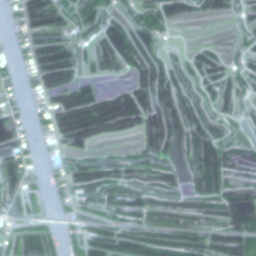

Supplement: S15 File — (ZIP) [file pone.0327315.s015.zip › INPUT_DONE/train_images/train/IMAGE_CaMau.tifpatch_1714.tif]

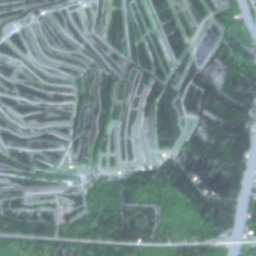

Supplement: S15 File — (ZIP) [file pone.0327315.s015.zip › INPUT_DONE/train_images/train/IMAGE_CaMau.tifpatch_1715.tif]

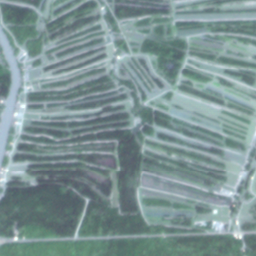

Supplement: S15 File — (ZIP) [file pone.0327315.s015.zip › INPUT_DONE/train_images/train/IMAGE_CaMau.tifpatch_1716.tif]

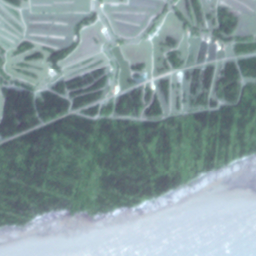

Supplement: S15 File — (ZIP) [file pone.0327315.s015.zip › INPUT_DONE/train_images/train/IMAGE_CaMau.tifpatch_1718.tif]

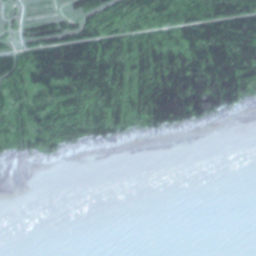

Supplement: S15 File — (ZIP) [file pone.0327315.s015.zip › INPUT_DONE/train_images/train/IMAGE_CaMau.tifpatch_1719.tif]

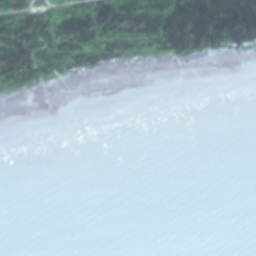

Supplement: S15 File — (ZIP) [file pone.0327315.s015.zip › INPUT_DONE/train_images/train/IMAGE_CaMau.tifpatch_1720.tif]

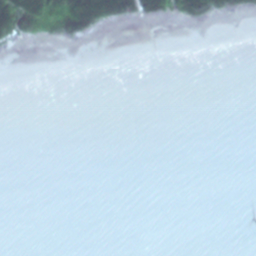

Supplement: S15 File — (ZIP) [file pone.0327315.s015.zip › INPUT_DONE/train_images/train/IMAGE_CaMau.tifpatch_1721.tif]

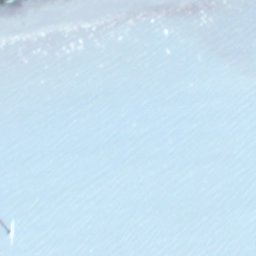

Supplement: S15 File — (ZIP) [file pone.0327315.s015.zip › INPUT_DONE/train_images/train/IMAGE_CaMau.tifpatch_1722.tif]

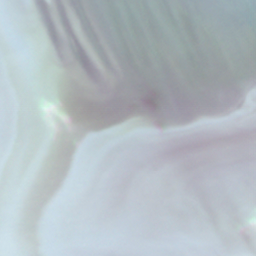

Supplement: S15 File — (ZIP) [file pone.0327315.s015.zip › INPUT_DONE/train_images/train/IMAGE_CaMau.tifpatch_176.tif]

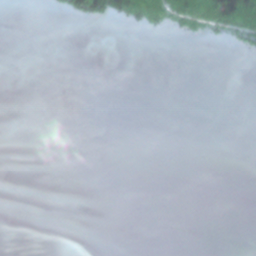

Supplement: S15 File — (ZIP) [file pone.0327315.s015.zip › INPUT_DONE/train_images/train/IMAGE_CaMau.tifpatch_178.tif]

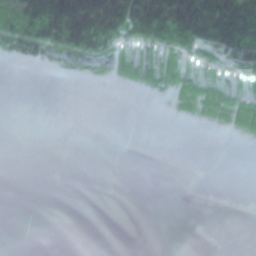

Supplement: S15 File — (ZIP) [file pone.0327315.s015.zip › INPUT_DONE/train_images/train/IMAGE_CaMau.tifpatch_179.tif]

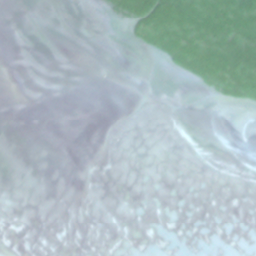

Supplement: S15 File — (ZIP) [file pone.0327315.s015.zip › INPUT_DONE/train_images/train/IMAGE_CaMau.tifpatch_1810.tif]

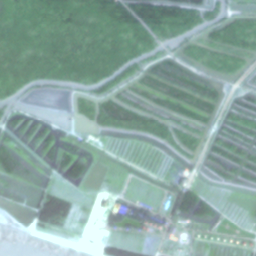

Supplement: S15 File — (ZIP) [file pone.0327315.s015.zip › INPUT_DONE/train_images/train/IMAGE_CaMau.tifpatch_1812.tif]
