# Supplementary figures and images for: Mangrove species classification using a proposed ensemble U-Net model and Planet satellite imagery: A case study in Ngoc Hien district, Ca Mau province, Vietnam (part 1 of 2)
Source: PLoS One. 2025 Aug 6;20(8):e0327315. doi: 10.1371/journal.pone.0327315 (PMC12327635; doi:10.1371/journal.pone.0327315)

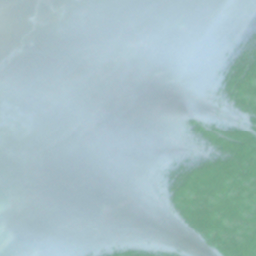

Supplement: S15 File — (ZIP) [file pone.0327315.s015.zip › INPUT_DONE/train_images/train/IMAGE_CaMau.tifpatch_016.tif]

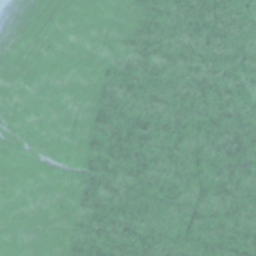

Supplement: S15 File — (ZIP) [file pone.0327315.s015.zip › INPUT_DONE/train_images/train/IMAGE_CaMau.tifpatch_017.tif]

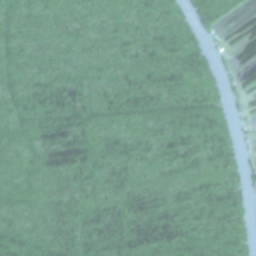

Supplement: S15 File — (ZIP) [file pone.0327315.s015.zip › INPUT_DONE/train_images/train/IMAGE_CaMau.tifpatch_018.tif]

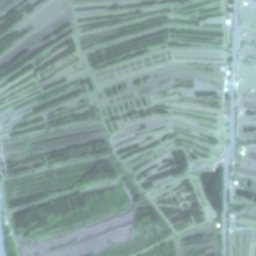

Supplement: S15 File — (ZIP) [file pone.0327315.s015.zip › INPUT_DONE/train_images/train/IMAGE_CaMau.tifpatch_019.tif]

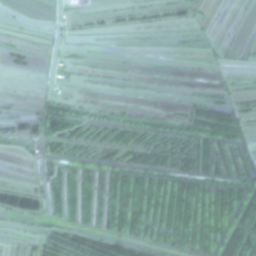

Supplement: S15 File — (ZIP) [file pone.0327315.s015.zip › INPUT_DONE/train_images/train/IMAGE_CaMau.tifpatch_020.tif]

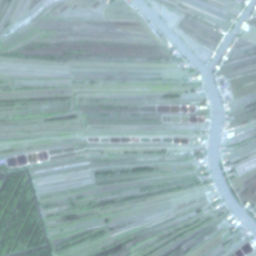

Supplement: S15 File — (ZIP) [file pone.0327315.s015.zip › INPUT_DONE/train_images/train/IMAGE_CaMau.tifpatch_021.tif]

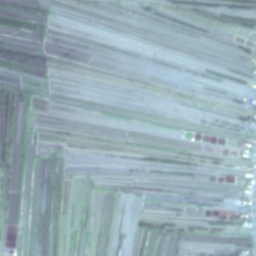

Supplement: S15 File — (ZIP) [file pone.0327315.s015.zip › INPUT_DONE/train_images/train/IMAGE_CaMau.tifpatch_023.tif]

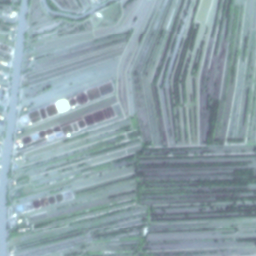

Supplement: S15 File — (ZIP) [file pone.0327315.s015.zip › INPUT_DONE/train_images/train/IMAGE_CaMau.tifpatch_024.tif]

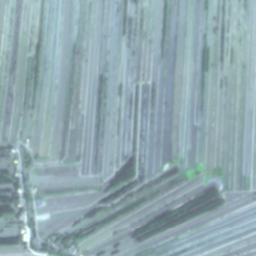

Supplement: S15 File — (ZIP) [file pone.0327315.s015.zip › INPUT_DONE/train_images/train/IMAGE_CaMau.tifpatch_025.tif]

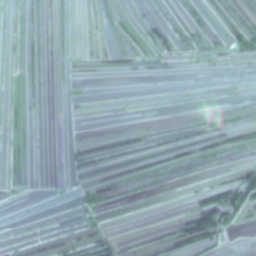

Supplement: S15 File — (ZIP) [file pone.0327315.s015.zip › INPUT_DONE/train_images/train/IMAGE_CaMau.tifpatch_026.tif]

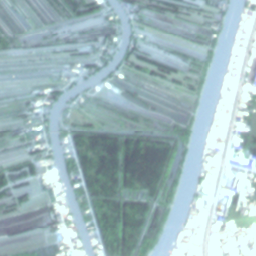

Supplement: S15 File — (ZIP) [file pone.0327315.s015.zip › INPUT_DONE/train_images/train/IMAGE_CaMau.tifpatch_027.tif]

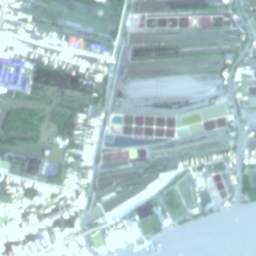

Supplement: S15 File — (ZIP) [file pone.0327315.s015.zip › INPUT_DONE/train_images/train/IMAGE_CaMau.tifpatch_028.tif]

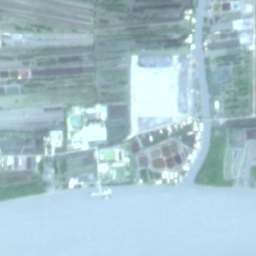

Supplement: S15 File — (ZIP) [file pone.0327315.s015.zip › INPUT_DONE/train_images/train/IMAGE_CaMau.tifpatch_029.tif]

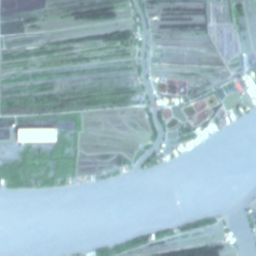

Supplement: S15 File — (ZIP) [file pone.0327315.s015.zip › INPUT_DONE/train_images/train/IMAGE_CaMau.tifpatch_030.tif]

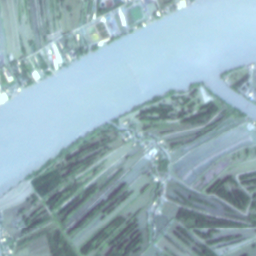

Supplement: S15 File — (ZIP) [file pone.0327315.s015.zip › INPUT_DONE/train_images/train/IMAGE_CaMau.tifpatch_031.tif]

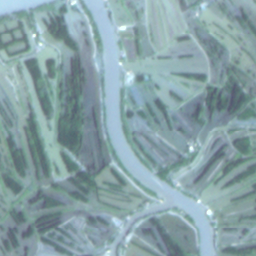

Supplement: S15 File — (ZIP) [file pone.0327315.s015.zip › INPUT_DONE/train_images/train/IMAGE_CaMau.tifpatch_033.tif]

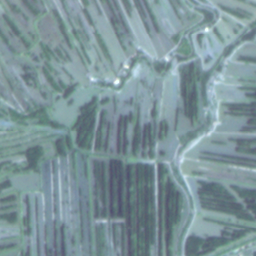

Supplement: S15 File — (ZIP) [file pone.0327315.s015.zip › INPUT_DONE/train_images/train/IMAGE_CaMau.tifpatch_034.tif]

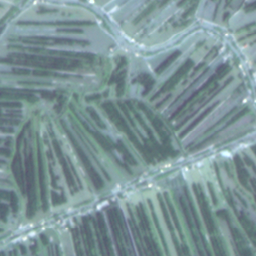

Supplement: S15 File — (ZIP) [file pone.0327315.s015.zip › INPUT_DONE/train_images/train/IMAGE_CaMau.tifpatch_035.tif]

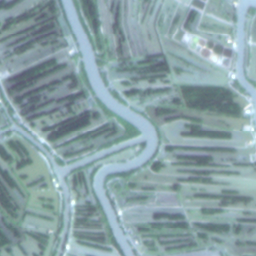

Supplement: S15 File — (ZIP) [file pone.0327315.s015.zip › INPUT_DONE/train_images/train/IMAGE_CaMau.tifpatch_036.tif]

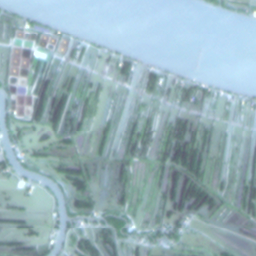

Supplement: S15 File — (ZIP) [file pone.0327315.s015.zip › INPUT_DONE/train_images/train/IMAGE_CaMau.tifpatch_037.tif]

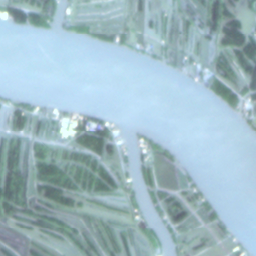

Supplement: S15 File — (ZIP) [file pone.0327315.s015.zip › INPUT_DONE/train_images/train/IMAGE_CaMau.tifpatch_038.tif]

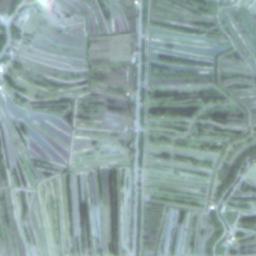

Supplement: S15 File — (ZIP) [file pone.0327315.s015.zip › INPUT_DONE/train_images/train/IMAGE_CaMau.tifpatch_040.tif]

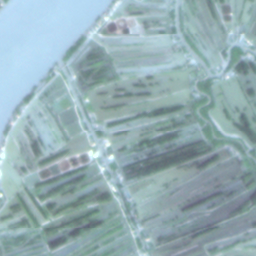

Supplement: S15 File — (ZIP) [file pone.0327315.s015.zip › INPUT_DONE/train_images/train/IMAGE_CaMau.tifpatch_042.tif]

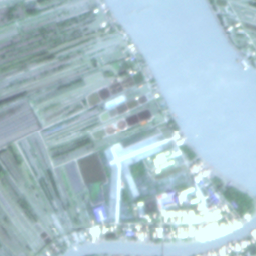

Supplement: S15 File — (ZIP) [file pone.0327315.s015.zip › INPUT_DONE/train_images/train/IMAGE_CaMau.tifpatch_045.tif]

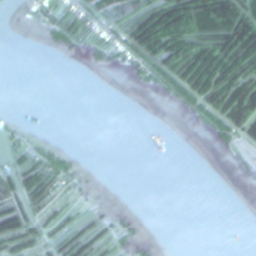

Supplement: S15 File — (ZIP) [file pone.0327315.s015.zip › INPUT_DONE/train_images/train/IMAGE_CaMau.tifpatch_047.tif]

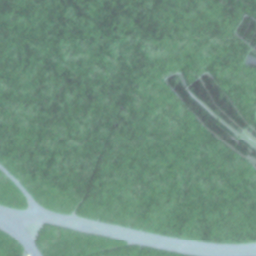

Supplement: S15 File — (ZIP) [file pone.0327315.s015.zip › INPUT_DONE/train_images/train/IMAGE_CaMau.tifpatch_1011.tif]

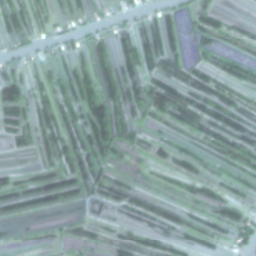

Supplement: S15 File — (ZIP) [file pone.0327315.s015.zip › INPUT_DONE/train_images/train/IMAGE_CaMau.tifpatch_1014.tif]

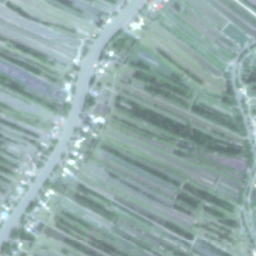

Supplement: S15 File — (ZIP) [file pone.0327315.s015.zip › INPUT_DONE/train_images/train/IMAGE_CaMau.tifpatch_1015.tif]

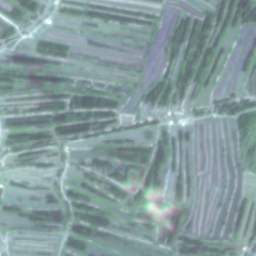

Supplement: S15 File — (ZIP) [file pone.0327315.s015.zip › INPUT_DONE/train_images/train/IMAGE_CaMau.tifpatch_1016.tif]

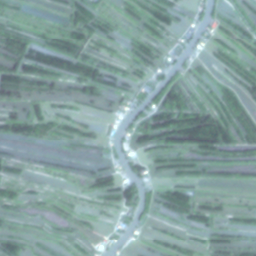

Supplement: S15 File — (ZIP) [file pone.0327315.s015.zip › INPUT_DONE/train_images/train/IMAGE_CaMau.tifpatch_1018.tif]

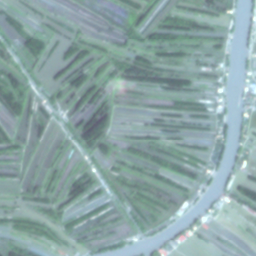

Supplement: S15 File — (ZIP) [file pone.0327315.s015.zip › INPUT_DONE/train_images/train/IMAGE_CaMau.tifpatch_1019.tif]

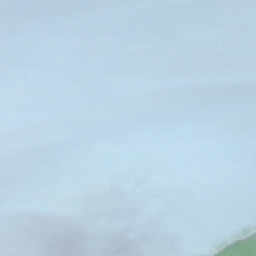

Supplement: S15 File — (ZIP) [file pone.0327315.s015.zip › INPUT_DONE/train_images/train/IMAGE_CaMau.tifpatch_102.tif]

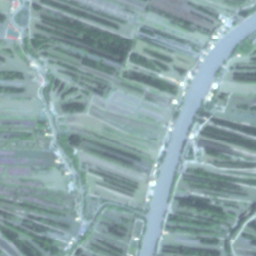

Supplement: S15 File — (ZIP) [file pone.0327315.s015.zip › INPUT_DONE/train_images/train/IMAGE_CaMau.tifpatch_1021.tif]

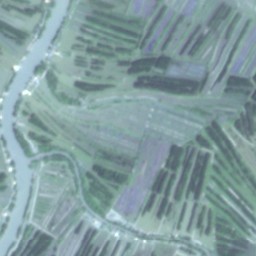

Supplement: S15 File — (ZIP) [file pone.0327315.s015.zip › INPUT_DONE/train_images/train/IMAGE_CaMau.tifpatch_1023.tif]

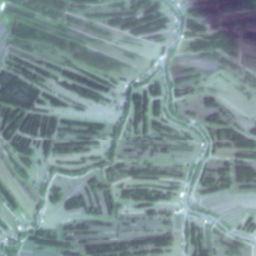

Supplement: S15 File — (ZIP) [file pone.0327315.s015.zip › INPUT_DONE/train_images/train/IMAGE_CaMau.tifpatch_1024.tif]

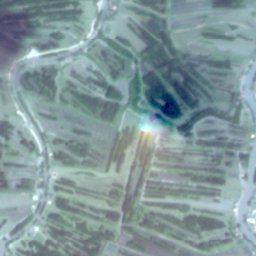

Supplement: S15 File — (ZIP) [file pone.0327315.s015.zip › INPUT_DONE/train_images/train/IMAGE_CaMau.tifpatch_1025.tif]

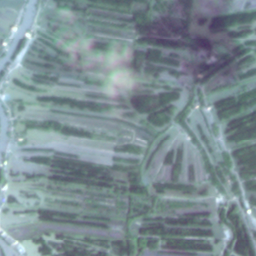

Supplement: S15 File — (ZIP) [file pone.0327315.s015.zip › INPUT_DONE/train_images/train/IMAGE_CaMau.tifpatch_1026.tif]

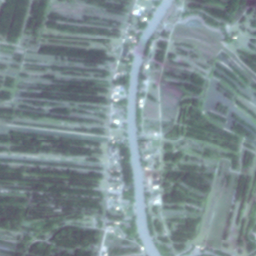

Supplement: S15 File — (ZIP) [file pone.0327315.s015.zip › INPUT_DONE/train_images/train/IMAGE_CaMau.tifpatch_1028.tif]

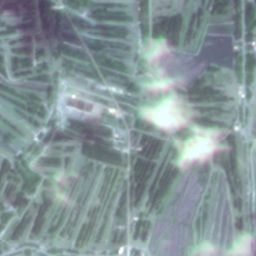

Supplement: S15 File — (ZIP) [file pone.0327315.s015.zip › INPUT_DONE/train_images/train/IMAGE_CaMau.tifpatch_1029.tif]

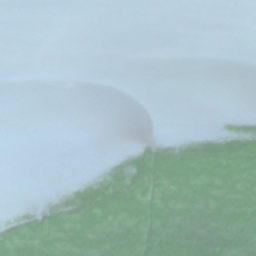

Supplement: S15 File — (ZIP) [file pone.0327315.s015.zip › INPUT_DONE/train_images/train/IMAGE_CaMau.tifpatch_103.tif]

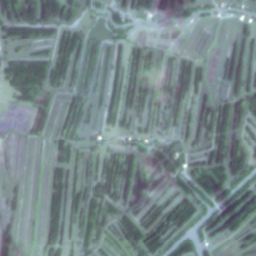

Supplement: S15 File — (ZIP) [file pone.0327315.s015.zip › INPUT_DONE/train_images/train/IMAGE_CaMau.tifpatch_1030.tif]

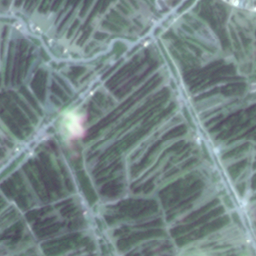

Supplement: S15 File — (ZIP) [file pone.0327315.s015.zip › INPUT_DONE/train_images/train/IMAGE_CaMau.tifpatch_1031.tif]

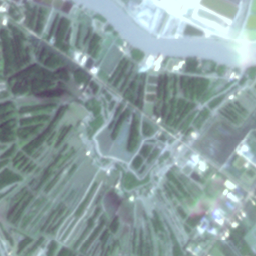

Supplement: S15 File — (ZIP) [file pone.0327315.s015.zip › INPUT_DONE/train_images/train/IMAGE_CaMau.tifpatch_1032.tif]

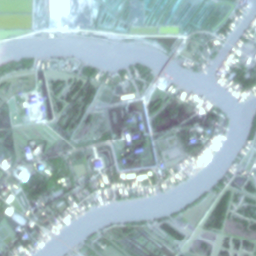

Supplement: S15 File — (ZIP) [file pone.0327315.s015.zip › INPUT_DONE/train_images/train/IMAGE_CaMau.tifpatch_1033.tif]

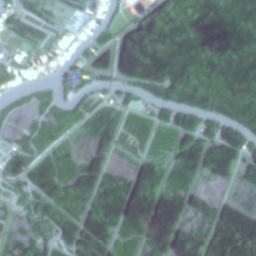

Supplement: S15 File — (ZIP) [file pone.0327315.s015.zip › INPUT_DONE/train_images/train/IMAGE_CaMau.tifpatch_1034.tif]

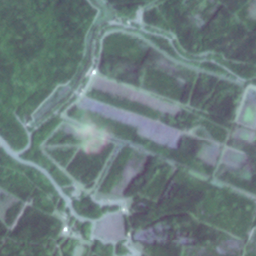

Supplement: S15 File — (ZIP) [file pone.0327315.s015.zip › INPUT_DONE/train_images/train/IMAGE_CaMau.tifpatch_1035.tif]

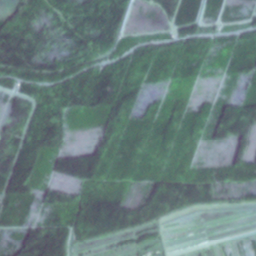

Supplement: S15 File — (ZIP) [file pone.0327315.s015.zip › INPUT_DONE/train_images/train/IMAGE_CaMau.tifpatch_1036.tif]

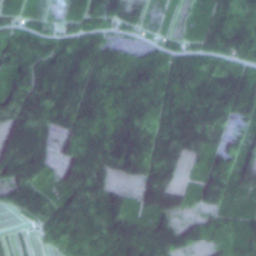

Supplement: S15 File — (ZIP) [file pone.0327315.s015.zip › INPUT_DONE/train_images/train/IMAGE_CaMau.tifpatch_1037.tif]

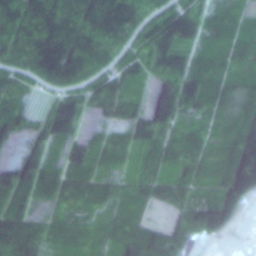

Supplement: S15 File — (ZIP) [file pone.0327315.s015.zip › INPUT_DONE/train_images/train/IMAGE_CaMau.tifpatch_1038.tif]

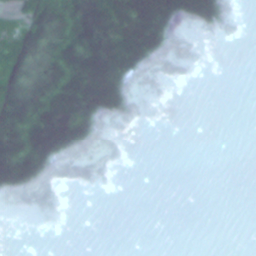

Supplement: S15 File — (ZIP) [file pone.0327315.s015.zip › INPUT_DONE/train_images/train/IMAGE_CaMau.tifpatch_1039.tif]

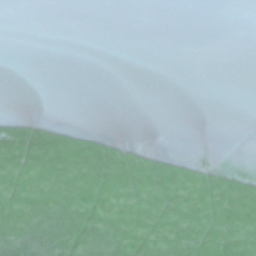

Supplement: S15 File — (ZIP) [file pone.0327315.s015.zip › INPUT_DONE/train_images/train/IMAGE_CaMau.tifpatch_104.tif]

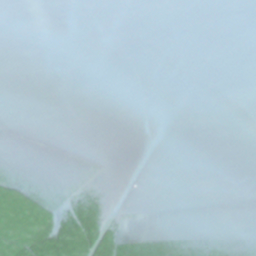

Supplement: S15 File — (ZIP) [file pone.0327315.s015.zip › INPUT_DONE/train_images/train/IMAGE_CaMau.tifpatch_105.tif]

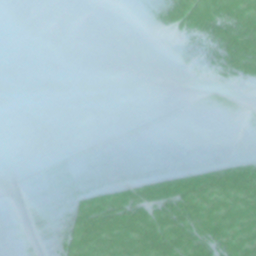

Supplement: S15 File — (ZIP) [file pone.0327315.s015.zip › INPUT_DONE/train_images/train/IMAGE_CaMau.tifpatch_108.tif]

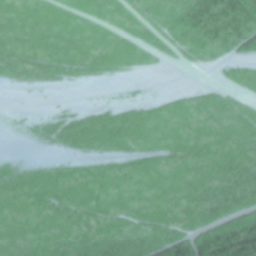

Supplement: S15 File — (ZIP) [file pone.0327315.s015.zip › INPUT_DONE/train_images/train/IMAGE_CaMau.tifpatch_109.tif]

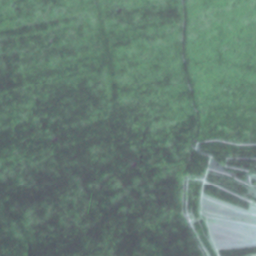

Supplement: S15 File — (ZIP) [file pone.0327315.s015.zip › INPUT_DONE/train_images/train/IMAGE_CaMau.tifpatch_1110.tif]

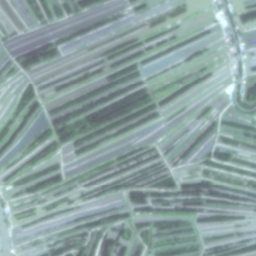

Supplement: S15 File — (ZIP) [file pone.0327315.s015.zip › INPUT_DONE/train_images/train/IMAGE_CaMau.tifpatch_1113.tif]

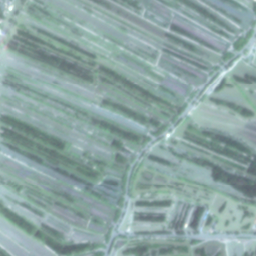

Supplement: S15 File — (ZIP) [file pone.0327315.s015.zip › INPUT_DONE/train_images/train/IMAGE_CaMau.tifpatch_1115.tif]

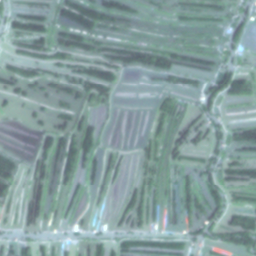

Supplement: S15 File — (ZIP) [file pone.0327315.s015.zip › INPUT_DONE/train_images/train/IMAGE_CaMau.tifpatch_1116.tif]

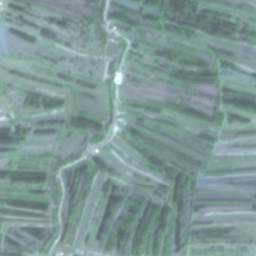

Supplement: S15 File — (ZIP) [file pone.0327315.s015.zip › INPUT_DONE/train_images/train/IMAGE_CaMau.tifpatch_1117.tif]

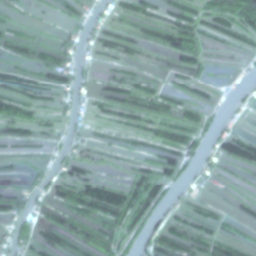

Supplement: S15 File — (ZIP) [file pone.0327315.s015.zip › INPUT_DONE/train_images/train/IMAGE_CaMau.tifpatch_1118.tif]

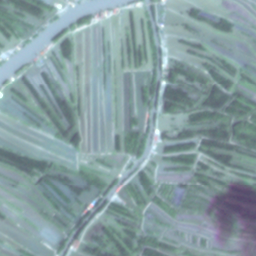

Supplement: S15 File — (ZIP) [file pone.0327315.s015.zip › INPUT_DONE/train_images/train/IMAGE_CaMau.tifpatch_1119.tif]

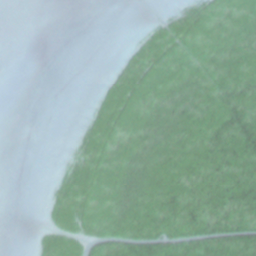

Supplement: S15 File — (ZIP) [file pone.0327315.s015.zip › INPUT_DONE/train_images/train/IMAGE_CaMau.tifpatch_112.tif]

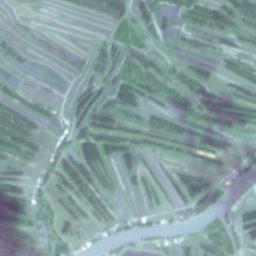

Supplement: S15 File — (ZIP) [file pone.0327315.s015.zip › INPUT_DONE/train_images/train/IMAGE_CaMau.tifpatch_1120.tif]

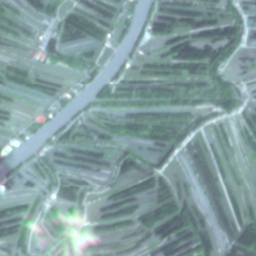

Supplement: S15 File — (ZIP) [file pone.0327315.s015.zip › INPUT_DONE/train_images/train/IMAGE_CaMau.tifpatch_1121.tif]

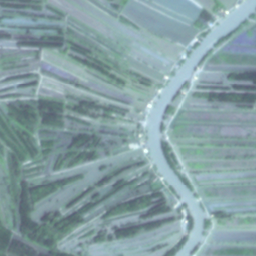

Supplement: S15 File — (ZIP) [file pone.0327315.s015.zip › INPUT_DONE/train_images/train/IMAGE_CaMau.tifpatch_1122.tif]

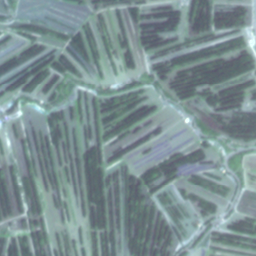

Supplement: S15 File — (ZIP) [file pone.0327315.s015.zip › INPUT_DONE/train_images/train/IMAGE_CaMau.tifpatch_1124.tif]

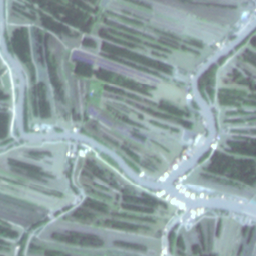

Supplement: S15 File — (ZIP) [file pone.0327315.s015.zip › INPUT_DONE/train_images/train/IMAGE_CaMau.tifpatch_1125.tif]

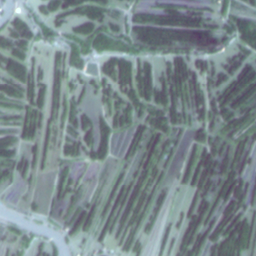

Supplement: S15 File — (ZIP) [file pone.0327315.s015.zip › INPUT_DONE/train_images/train/IMAGE_CaMau.tifpatch_1126.tif]

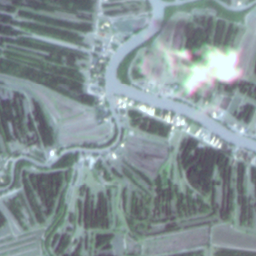

Supplement: S15 File — (ZIP) [file pone.0327315.s015.zip › INPUT_DONE/train_images/train/IMAGE_CaMau.tifpatch_1128.tif]

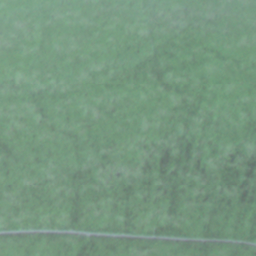

Supplement: S15 File — (ZIP) [file pone.0327315.s015.zip › INPUT_DONE/train_images/train/IMAGE_CaMau.tifpatch_113.tif]

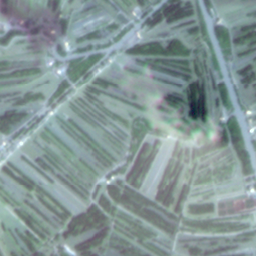

Supplement: S15 File — (ZIP) [file pone.0327315.s015.zip › INPUT_DONE/train_images/train/IMAGE_CaMau.tifpatch_1130.tif]

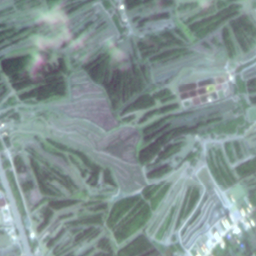

Supplement: S15 File — (ZIP) [file pone.0327315.s015.zip › INPUT_DONE/train_images/train/IMAGE_CaMau.tifpatch_1131.tif]

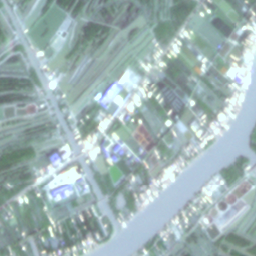

Supplement: S15 File — (ZIP) [file pone.0327315.s015.zip › INPUT_DONE/train_images/train/IMAGE_CaMau.tifpatch_1132.tif]

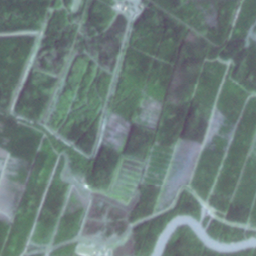

Supplement: S15 File — (ZIP) [file pone.0327315.s015.zip › INPUT_DONE/train_images/train/IMAGE_CaMau.tifpatch_1135.tif]

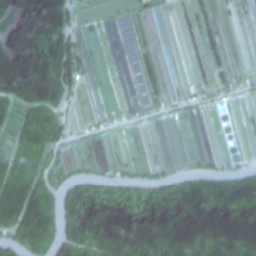

Supplement: S15 File — (ZIP) [file pone.0327315.s015.zip › INPUT_DONE/train_images/train/IMAGE_CaMau.tifpatch_1136.tif]

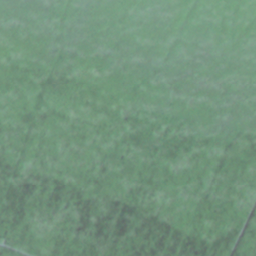

Supplement: S15 File — (ZIP) [file pone.0327315.s015.zip › INPUT_DONE/train_images/train/IMAGE_CaMau.tifpatch_114.tif]

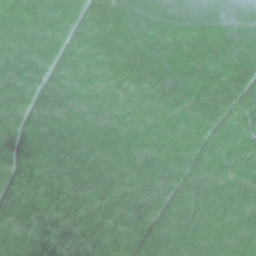

Supplement: S15 File — (ZIP) [file pone.0327315.s015.zip › INPUT_DONE/train_images/train/IMAGE_CaMau.tifpatch_115.tif]

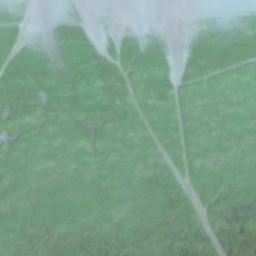

Supplement: S15 File — (ZIP) [file pone.0327315.s015.zip › INPUT_DONE/train_images/train/IMAGE_CaMau.tifpatch_116.tif]

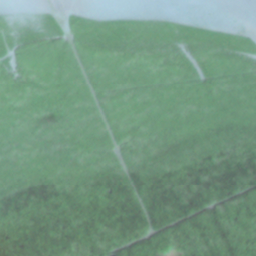

Supplement: S15 File — (ZIP) [file pone.0327315.s015.zip › INPUT_DONE/train_images/train/IMAGE_CaMau.tifpatch_117.tif]

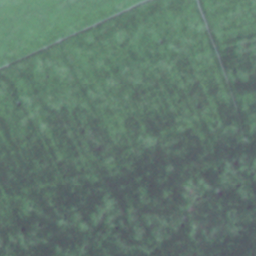

Supplement: S15 File — (ZIP) [file pone.0327315.s015.zip › INPUT_DONE/train_images/train/IMAGE_CaMau.tifpatch_119.tif]

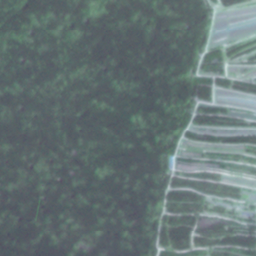

Supplement: S15 File — (ZIP) [file pone.0327315.s015.zip › INPUT_DONE/train_images/train/IMAGE_CaMau.tifpatch_1210.tif]

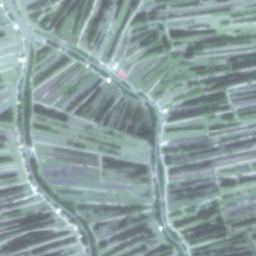

Supplement: S15 File — (ZIP) [file pone.0327315.s015.zip › INPUT_DONE/train_images/train/IMAGE_CaMau.tifpatch_1213.tif]

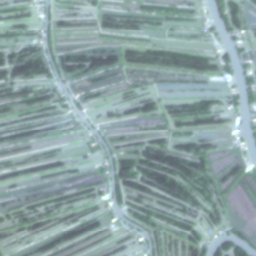

Supplement: S15 File — (ZIP) [file pone.0327315.s015.zip › INPUT_DONE/train_images/train/IMAGE_CaMau.tifpatch_1214.tif]

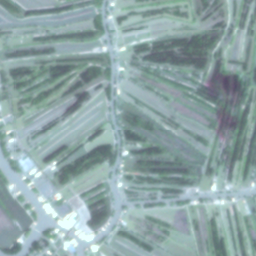

Supplement: S15 File — (ZIP) [file pone.0327315.s015.zip › INPUT_DONE/train_images/train/IMAGE_CaMau.tifpatch_1215.tif]

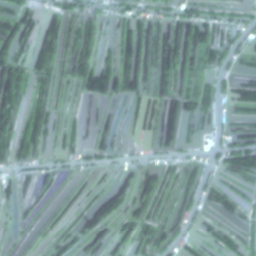

Supplement: S15 File — (ZIP) [file pone.0327315.s015.zip › INPUT_DONE/train_images/train/IMAGE_CaMau.tifpatch_1217.tif]

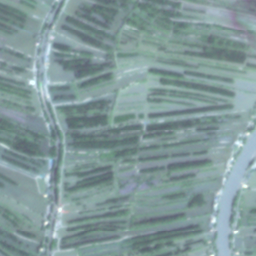

Supplement: S15 File — (ZIP) [file pone.0327315.s015.zip › INPUT_DONE/train_images/train/IMAGE_CaMau.tifpatch_1219.tif]

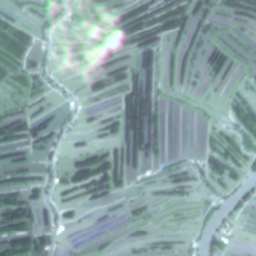

Supplement: S15 File — (ZIP) [file pone.0327315.s015.zip › INPUT_DONE/train_images/train/IMAGE_CaMau.tifpatch_1221.tif]

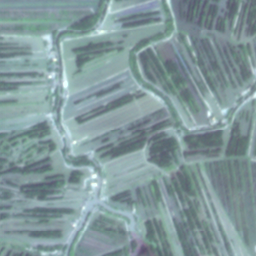

Supplement: S15 File — (ZIP) [file pone.0327315.s015.zip › INPUT_DONE/train_images/train/IMAGE_CaMau.tifpatch_1223.tif]

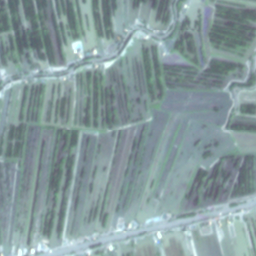

Supplement: S15 File — (ZIP) [file pone.0327315.s015.zip › INPUT_DONE/train_images/train/IMAGE_CaMau.tifpatch_1224.tif]

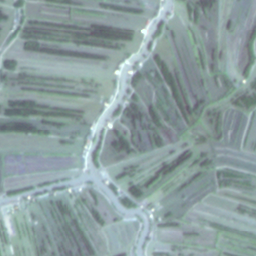

Supplement: S15 File — (ZIP) [file pone.0327315.s015.zip › INPUT_DONE/train_images/train/IMAGE_CaMau.tifpatch_1225.tif]

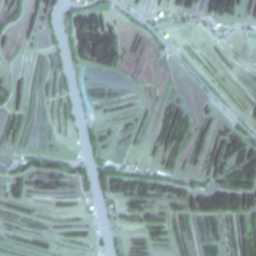

Supplement: S15 File — (ZIP) [file pone.0327315.s015.zip › INPUT_DONE/train_images/train/IMAGE_CaMau.tifpatch_1226.tif]

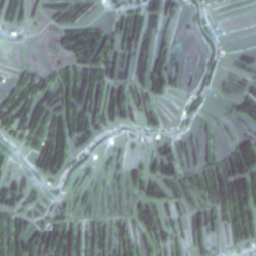

Supplement: S15 File — (ZIP) [file pone.0327315.s015.zip › INPUT_DONE/train_images/train/IMAGE_CaMau.tifpatch_1227.tif]

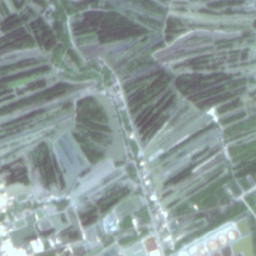

Supplement: S15 File — (ZIP) [file pone.0327315.s015.zip › INPUT_DONE/train_images/train/IMAGE_CaMau.tifpatch_1230.tif]

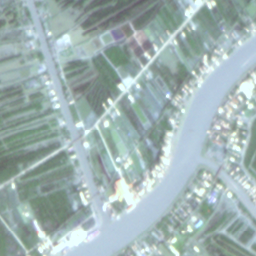

Supplement: S15 File — (ZIP) [file pone.0327315.s015.zip › INPUT_DONE/train_images/train/IMAGE_CaMau.tifpatch_1231.tif]

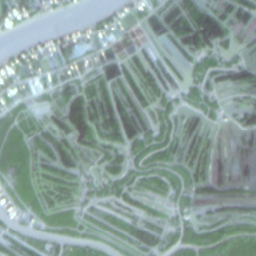

Supplement: S15 File — (ZIP) [file pone.0327315.s015.zip › INPUT_DONE/train_images/train/IMAGE_CaMau.tifpatch_1232.tif]

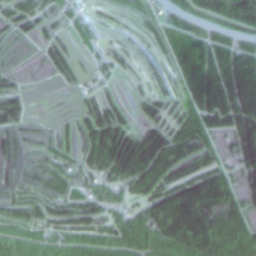

Supplement: S15 File — (ZIP) [file pone.0327315.s015.zip › INPUT_DONE/train_images/train/IMAGE_CaMau.tifpatch_1233.tif]

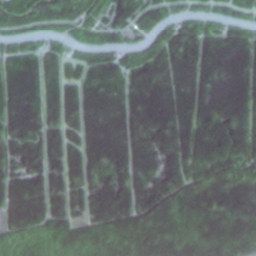

Supplement: S15 File — (ZIP) [file pone.0327315.s015.zip › INPUT_DONE/train_images/train/IMAGE_CaMau.tifpatch_1234.tif]

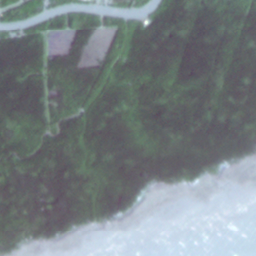

Supplement: S15 File — (ZIP) [file pone.0327315.s015.zip › INPUT_DONE/train_images/train/IMAGE_CaMau.tifpatch_1235.tif]

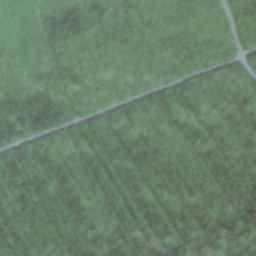

Supplement: S15 File — (ZIP) [file pone.0327315.s015.zip › INPUT_DONE/train_images/train/IMAGE_CaMau.tifpatch_126.tif]

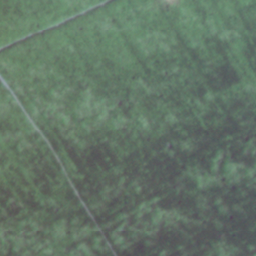

Supplement: S15 File — (ZIP) [file pone.0327315.s015.zip › INPUT_DONE/train_images/train/IMAGE_CaMau.tifpatch_127.tif]
