# Supplementary material for: Mangrove species classification using a proposed ensemble U-Net model and Planet satellite imagery: A case study in Ngoc Hien district, Ca Mau province, Vietnam
Source: PLoS One. 2025 Aug 6;20(8):e0327315. doi: 10.1371/journal.pone.0327315 (PMC12327635; doi:10.1371/journal.pone.0327315)
Supplement: Attachment — Submitted filename: Review_2.docx [file pone.0327315.s016.docx]

Plant species identification using satellite images has been a widely addressed area of research. The novelty of this research is addressing a specific species, Mangrove, as a case study in Vietnam.
The following improvements are suggested.
1. It would be better to highlight the main research questions in the introduction section, and describe in the Discussion section, how you fulfilled those in the study, by referring to the applied methods and the obtained results.

2. Include a new section 2 for related studies. it would be better to consider ML based land use identification using satellite images published in recent years with the latest technologies. Discuss the techniques used in the related studies on land cover identification such as https://doi.org/10.1145/3056662.3056681 and https://doi.org/10.1002/9781119682042.ch1 together with the limitations in the existing studies and justify the proposed method.

You can consider other cropland identification work such as https://doi.org/10.1016/B978-0-323-85214-2.00009-4 and https://doi.org/10.1016/B978-0-323-90550-3.00008-4

3. Clearly describe the novel scientific contribution. There are many U-net types available in the literature. Justify the selection of your approach with ensemble U-Net.
4. Your scientific contribution is sufficient. However, you need to justify the contributions.
5. Most of the figures are not clear. You have to refine the visualization aspects of your study.

6. Clearly state the used datasets and the number of images for different labels, before augmentation and the data fed to the training process. – That is, the considering the two labels buildings/ background, the number of images in each class for training, testing and validation sets. Discuss data balancing/ imbalancing.

7. Have you check the generalizability of the proposed model, for different datasets.

8. Consider the results graphs/ figures. Instead of stating as Fig. x shows…it would be better to explain the observations that can be seen in the graph, and discuss the conclusion that can be made from those graph observations. This will help readers to identify the importance of the findings.

9. In the discussion section, justify the achievements of the said contributions or research questions mentioned in the introduction, referring to the followed methodology and the obtained results.

10. Compare the results of this study with latest related work.


11. In the discussion, state the research limitations and the future possible extensions.
12. Discuss the possible practical applications, of this model.
