# Supplementary material for: Mangrove species classification using a proposed ensemble U-Net model and Planet satellite imagery: A case study in Ngoc Hien district, Ca Mau province, Vietnam
Source: PLoS One. 2025 Aug 6;20(8):e0327315. doi: 10.1371/journal.pone.0327315 (PMC12327635; doi:10.1371/journal.pone.0327315)
Supplement: Attachment — Submitted filename: Review_1.docx [file pone.0327315.s017.docx]

The manuscript titled “Mangrove species classification using a proposed ensemble U-Net model and Planet satellite imagery: A case study in Ngoc Hien district, Ca Mau province, Vietnam” presents an interesting work on Mangrove species classification. However, the manuscript can be further improved in respect of the following points:

1. In line 28 on page 2, it should be “Intersection over Union”.
2. The literature survey can be improved focusing particularly on the work carried out for Mangrove species classification.
3. There is no mention about how the ground truth data was obtained.
4. Experiments can be carried out to prove the point of using a combination of Focal loss and Dice loss by assessing the impact of employing only the Focal loss or the Dice loss on the classification results. These results can be compared to those achieved using the combination of Focal loss and Dice loss.
5. What about the computational complexity of the models employed?
6. The quality of Figures 3, 4, and 5 needs to be improved.
7. In Table 2, the results for only four models, i. e., M3, M10, M12, and EM were shown, what about the other models?
