# Supplementary material for: Mangrove species classification using a proposed ensemble U-Net model and Planet satellite imagery: A case study in Ngoc Hien district, Ca Mau province, Vietnam
Source: PLoS One. 2025 Aug 6;20(8):e0327315. doi: 10.1371/journal.pone.0327315 (PMC12327635; doi:10.1371/journal.pone.0327315)
Supplement: Attachment — Submitted filename: Response to Reviewers.docx [file pone.0327315.s019.docx]

Dear Academic Editor and Reviewers,

Thank you very much for your valuable comments. After carefully revising the manuscript, I agreed with your comments, which were effective in helping us revise the manuscript to meet PLOS ONE's publication criteria as it currently stands. I have carefully revised and prepared the additional documents, such as: Input dataset, codes and results of the trained U-Net models, and testing losses (attached to this letter).

In this letter, I would like to respond to each point raised by the academic editor and two reviewers during the review process, including:

Part 1. Responses to Academic Editor;

Part 2. Responses to Reviewer #1;

Part 3. Responses to Reviewer #2;

Best regards,

Tran Dang Hung

**Part 1. Responses to Academic Editor**

**Comment 1.** Please ensure that your manuscript meets PLOS ONE's style requirements, including those for file naming. The PLOS ONE style templates can be found at <https://journals.plos.org/plosone/s/file?id=wjVg/PLOSOne_formatting_sample_main_body.pdf>  and [https://journals.plos.org/plosone/s/file?id=ba62/PLOSOne_formatting_sample_title_authors_affiliations.pdf](https://track.editorialmanager.com/CL0/https:%2F%2Fjournals.plos.org%2Fplosone%2Fs%2Ffile%3Fid=ba62%2FPLOSOne_formatting_sample_title_authors_affiliations.pdf/1/010f01945ebab695-438e4e78-5e5c-4677-8d09-be3a34079ca8-000000/BfS95v9fLBI1oz8VXmMLyYnFZXtnWXXaomObVUVG74Y=193)

***Response***: We checked the manuscript and corrected it following PLOS ONE's style requirements, including those for file naming mentioned in the links: <https://journals.plos.org/plosone/s/file?id=wjVg/PLOSOne_formatting_sample_main_body.pdf>  and [https://journals.plos.org/plosone/s/file?id=ba62/PLOSOne_formatting_sample_title_authors_affiliations.pdf](https://track.editorialmanager.com/CL0/https:%2F%2Fjournals.plos.org%2Fplosone%2Fs%2Ffile%3Fid=ba62%2FPLOSOne_formatting_sample_title_authors_affiliations.pdf/1/010f01945ebab695-438e4e78-5e5c-4677-8d09-be3a34079ca8-000000/BfS95v9fLBI1oz8VXmMLyYnFZXtnWXXaomObVUVG74Y=193)

**Comment 2**. We note that your Data Availability Statement is currently as follows: [All relevant data are within the manuscript and its Supporting Information files.] Please confirm at this time whether or not your submission contains all raw data required to replicate the results of your study. Authors must share the “minimal data set” for their submission. PLOS defines the minimal data set to consist of the data required to replicate all study findings reported in the article, as well as related metadata and methods (<https://journals.plos.org/plosone/s/data-availability#loc-minimal-data-set-definition>).

***Response***: We have additionally provided all of our used data and codes, including:

+) Input dataset used (“Input_Dataset”)

+) Codes and results of twelve trained single U-Net models (“SM”)

+) Code and results of our proposed ensemble U-Net model (“EM”)

+) Code and results for testing losses (“Test_Losses”)

At this time, we confirm that our submission contains all raw data required to replicate the results of our study.

**Comment** **3.** Please ensure that you refer to Figure 2 in your text as, if accepted, production will need this reference to link the reader to the figure.

***Response:*** Figure 2, which was used to supply information for the used dataset, was referred in the "Dataset" section (Lines 151-152). In particular, the dataset included two images: an original image (a Planet satellite image) and a labelled image (a classified land-cover image) (Fig 2).

**Part 2. Responses to Reviewer #1**

**Comment** **1.** In line 28 on page 2, it should be “Intersection over Union”.

***Response:*** We have checked and adjusted it to be "Intersection over Union" (Line 32).

**Comment** **2.** The literature survey can be improved focusing particularly on the work carried out for Mangrove species classification.

***Response:*** Following your suggestion, to better focus on the research objective, we have added more previous studies on mangrove classification based on traditional classification methods and machine learning approaches at the beginning of paragraph 2 of the "Introduction" section (Lines 59-67). Besides, this is the first study applying deep learning to classify land cover comprising mangrove species, therefore no previous studies exist.

**Comment 3.** There is no mention about how the ground truth data was obtained.

***Response:*** The implementation of the ground truth data was provided additionally in the "Dataset" section (Lines 156-159). In particular, the ground truth data was collected in June 2022, corresponding to the period the original image was downloaded, and described land cover at 100 randomly selected reference points that contained 70 points of mangrove forest. The labelled image was developed based on our classified land-cover map that was conducted using data from Planet imagery and ground truth.

**Comment** **4.** Experiments can be carried out to prove the point of using a combination of Focal loss and Dice loss by assessing the impact of employing only the Focal loss or the Dice loss on the classification results. These results can be compared to those achieved using the combination of Focal loss and Dice loss.

***Response:*** Based on your valuable suggestion, the experiments to assess the impact of employing only the Focal loss or Dice loss and the combination of Focal loss and Dice loss were conducted. The results were presented additionally in Table 2 below. Case (3) marked an outstanding identification for all classes, which gained the highest quantitative assessment metrics. (Lines 210-213, lines 224-225, and lines 634-635)

| **Classes** | **Metrics (%)** | **Models M3** | | |
| --- | --- | --- | --- | --- |
|  |  | **Focal loss** | **Dice loss** | **Focal loss**  **and Dice loss** |
| All classes | IoU | 75.59 | 74.54 | 76.63 |
|  | F1-score | 85.26 | 84.74 | 85.91 |
|  | OA | 92.21 | 90.66 | 93.17 |

**Comment 5.** What about the computational complexity of the models employed?

***Response:*** The number of parameters, depth, and GFLOPs affect the computation complexity of any single U-Net backbone model. All of these information was mentioned in Table 1. The higher the values of these criteria were, the more the computation complexity and time training increased. In each backbone type, each backbone with the highest computational complexity (the maximum number of parameters, depth layers, and GFLOPs) was selected to train with the promise of gaining the best classification result. (Lines 180-189, lines 273-276, and line 632)

**Comment** **6.** The quality of Figures 3, 4, and 5 needs to be improved.

***Response:*** The quality of Figures was improved from 300dpi to 600dpi.

**Comment** **7.** In Table 2, the results for only four models, i. e., M3, M10, M12, and EM were shown, what about the other models?

***Response:*** Sample classification results with different deep-learning U-Net backbone models were supplemented for all trained models (Lines 637-638). However, our study aimed at not only classifying mangrove species but also ensuring that the remaining classes were well classified, with high accuracy. Thereby, for the next step of developing an ensemble U-Net model, the single U-Net backbone model results were analyzed based on the assessment results of all classes. Then, Table 2 was used to compare the classification performance of the classes between the ensemble and the single-component models. (Lines 278-281)

**Part 3. Responses to Reviewer #2**

**Comment** **1.** It would be better to highlight the main research questions in the introduction section, and describe in the Discussion section, how you fulfilled those in the study, by referring to the applied methods and the obtained results.

***Responses:*** We clarified the main research objective in the last paragraph of the "Introduction" section and separated the parts in the "Discussion" section to answer the objective questions (Lines 121-124, lines 334-335, and line 379). The objectives of this study were: (1) to apply the superiority of deep learning for land cover comprising mangrove species classification over traditional classification methods, (2) to enhance classification performance and prediction efficiency of deep learning approach by a proposed ensemble U-Net model combining multiple single U-Net backbone models.

**Comment** **2.** Include a new section 2 for related studies, it would be better to consider ML based land use identification using satellite images published in recent years with the latest technologies. Discuss the techniques used in the related studies on land cover identification such as <https://doi.org/10.1145/3056662.3056681> and <https://doi.org/10.1002/9781119682042.ch1> together with the limitations in the existing studies and justify the proposed method.

You can consider other cropland identification work such as <https://doi.org/10.1016/B978-0-323-85214-2.00009-4> and <https://doi.org/10.1016/B978-0-323-90550-3.00008-4>

***Response:*** We have consulted the references you provided and added some related studies in paragraphs 3 and 4 of the "Introduction" section (such as the study of Barbosa et al. (2020) or Mahakalanda et al. (2022)) to further highlight the need for applying deep learning to mangrove species classification (Lines 89-97, and lines 111-115).

**Comment 3.** Clearly describe the novel scientific contribution. There are many U-net types available in the literature. Justify the selection of your approach with ensemble U-Net.

***Response:*** The explanation for this issue was supplemented in the "Deep learning segmentation using a U-Net model" part of the "Materials and methods" section (Lines 184-193). In this study, we used Google Colab to train the models, so the U-Net types in Google Colab were chosen. Additionally, to satisfy the objective of improving classification performance and prediction efficiency, in each backbone type, each backbone with the highest computational complexity (the maximum number of parameters, depth layers, and GFLOPs) was selected to train. In addition, if two backbones of the same type have the same values for one of the criteria (number of parameters, depth, or GFLOPs) or have equivalent values for all criteria, both backbones were considered for the experiment. Twelve U-Net backbone models selected were Model 1 (M1): ResNet-152; Model 2 (M2): SEResNet-152; Model 3 (M3): SEResNeXt-101; Model 4 (M4): SENet-154; Model 5 (M5): ResNeXt101; Model 6 (M6): VGG19; Model 7 (M7): DenseNet201; Model 8 (M8): InceptionResNetV2; Model 9 (M9): Inception-v3; Model 10 (M10): MobileNet; Model 11 (M11): MobileNet-v2; Model 12 (M12): EfficientNet-B7.

**Comment 4.** Your scientific contribution is sufficient. However, you need to justify the contributions.

***Response:*** We justified the contributions in the main objectives of the "Introduction" section and the "Discussion" section (Lines 121-130, and lines 334-454).

**Comment 5**. Most of the figures are not clear. You have to refine the visualization aspects of your study.

***Response:*** The quality of the figures was improved from 300dpi to 600dpi.

**Comment** **6.** Clearly state the used datasets and the number of images for different labels, before augmentation and the data fed to the training process. – That is, the considering the two labels buildings/ background, the number of images in each class for training, testing and validation sets. Discuss data balancing/ imbalancing.

***Response:*** This issue was presented additionally in the "Dataset" part (Lines 151-163) and discussed more in the “Proposed ensemble model for mangrove species classification” (Lines 420-425) and "Limitations and future extensions" (Lines 436-440) parts. Before augmentation, the dataset included two images: an original image (a Planet satellite image) and a labelled image (a classified land-cover image). After augmentation, 550 tiles (256x256) of the original image and 550 tiles (256x256) of the labelled image were created. Then, a 75:25 ratio was used to divide them randomly into training and validation datasets, in which the mangrove layers were encountered in 120 and 50 images, respectively. The dataset used was imbalanced, where six classes have different numbers of pixels (samples). This impacted the model's generalizability, particularly for less dominant mangrove species. The transfer deep learning techniques (the M10 model or our proposed ensemble U-Net model) could be employed to adapt to different geographic regions and improve its generalization to other mangrove areas.

**Comment** **7.** Have you check the generalizability of the proposed model, for different datasets.

***Response:*** Due to current funding constraints, checking the generalizability of the proposed model for another region is not possible. We have also highlighted this in the "Limitations and future extensions" part added to the "Discussion” section and proposed a solution for the future (Lines 436-440 and lines 450-452).

**Comment** **8.** Consider the results graphs/ figures. Instead of stating as Fig. x shows…it would be better to explain the observations that can be seen in the graph, and discuss the conclusion that can be made from those graph observations. This will help readers to identify the importance of the findings.

***Response:*** Based on your suggestion, our writing was improved in the hope of helping readers recognize the importance of the findings, especially in the "Research results” section (Lines 270-271, lines 281-283, and lines 318-319).

**Comment** **9.** In the discussion section, justify the achievements of the said contributions or research questions mentioned in the introduction, referring to the followed methodology and the obtained results.

***Response:*** We separated the parts in the "Discussion" section to clarify and answer the objective questions. There were three parts, including “Single U-Net backbone model vs conventional classification method for mangrove classification”, “Proposed ensemble model for mangrove species classification”, and “Limitations and future extensions.”

**Comment** **10.** Compare the results of this study with latest related work.

***Response:*** In the "Discussion" section, this study was compared with the latest studies. For example, the study of Ahad et al. (2023) confirmed that the choice of SEResNeXt101 (M3) for building the ensemble model is completely reasonable because of this model's outstanding classification advantages. Our result was consistent with that of Sevi and Aydin (2023) in detecting railway lines by applying U-Net segmentation performance using an ensemble model. When our experiment suggested that the EM combined the optimum characteristics of the single-component models, the classification accuracy rose by 3%, and the consistency of the classes' accuracy improved. (Lines 391-393 and lines 409-412)

**Comment** **11.** In the discussion, state the research limitations and the future possible extensions.

***Response:*** We have added a "Limitations and future extensions" part in the "Discussion" section to elaborate on these points (Line 430-454).

**Comment** **12.** Discuss the possible practical applications, of this model.

***Response:*** The "Introduction" section and the "Limitations and future extensions" part added to the "Discussion" section also presented practical applications that can be implemented. The transfer deep learning techniques (the M10 model or our proposed ensemble U-Net model) could be employed to adapt for different geographic regions, assisting managers and ecological planners by providing precise and timely data, improving the efficiency of land-cover monitoring, and preserving the long-term sustainability of mangrove forests. (Lines 450-454, lines 121-124, and lines 126-130)
