# Supplementary material for: Mangrove species classification using a proposed ensemble U-Net model and Planet satellite imagery: A case study in Ngoc Hien district, Ca Mau province, Vietnam
Source: PLoS One. 2025 Aug 6;20(8):e0327315. doi: 10.1371/journal.pone.0327315 (PMC12327635; doi:10.1371/journal.pone.0327315)
Supplement: Attachment — Submitted filename: Response_to_Reviewers_auresp_2.docx [file pone.0327315.s020.docx]

Dear Academic Editor and Reviewers,

We sincerely appreciate your contribution. After carefully revising, we concurred with your comments and made the necessary revisions to ensure that our manuscript is in a better version and satisfies PLOS ONE's publishing requirements.

In this letter, we would like to respond to every concern highlighted by the academic editor and two reviewers throughout the review process, including:

Part 1. Responses to Academic Editor;

Part 2. Responses to Reviewer #2;

Part 3. Responses to Reviewer #3.

Best regards,

Tran Dang Hung

**Part 1. Responses to Academic Editor**

**Comment 1**. Please review your reference list to ensure that it is complete and correct. If you have cited papers that have been retracted, please include the rationale for doing so in the manuscript text, or remove these references and replace them with relevant current references. Any changes to the reference list should be mentioned in the rebuttal letter that accompanies your revised manuscript. If you need to cite a retracted article, indicate the article’s retracted status in the References list and also include a citation and full reference for the retraction notice.

***Response***: Thank you for your contribution. We have reviewed our reference list and are certain that it is complete, correct, and un-retracted. Compared to the previous manuscript, the two latest references of Ma et al. (2025) and Irfan et al. (2025) were added based on the comment of Reviewer#2 (Lines 602-603 and lines 621-622).

**Part 2. Responses to Reviewer #2**

**Comment 1**. Paper is improved. However, proofread well for the clarity and language improvements. Since this paper is going to publish in 2025, it would be better to include some more latest literature.

***Response:*** We would like to express our gratitude for your comment. Based on your suggestion, we added two latest literature for our manuscript in the “Single U-Net backbone model vs conventional classification method for mangrove classification” section (Lines 378-381) and the “Limitations and future extensions” section (Lines 453 - 456). Additionally, the reference list was also supplemented with reference numbers 41 and 50.

**Part 3. Responses to Reviewer #3**

**Comment** **1.** Line no. 154 Hyperlink to mention the exact source and full path

***Responses:*** Thank you for your comment. We have checked and mentioned the exact source and full path (Line 150). However, if you want to download satellite images using this path, you must first create an account.

**Comment** **2.** Line Nos. 181 & 182 Mentioned "execution that does not need any installations," required import package installations or not for execution. Rephrase it.

***Response:*** We would to give our thanks for your contribution. Your mention was rephrased to clarify the meaning (Lines 177-179).

**Comment 3.** Line No. 186. GFLOPs for the first time mentioning the paragraph extent to full-length abbreviation.

***Response***: Thanks for your contribution. GFLOPs was extended to a full-length abbreviation - “Giga Floating Point Operations per Second” (Lines 183-184).

**Comment 4.** Line no. 210 and no. 210, no. 210, no. 210, and no. 210, no. 210, no.210,211 mentioned (1), (2), and (3) about table numbering or metric equation numbering not being addressed/referred to inside the paragraph text before or after the numbering with statement explanation.

***Response:*** Thanks for your comment. The table numbering or metric equation numbering was referred to inside the paragraph text before or after the numbering with a statement explanation (Line 176, lines 208-211, line 219, lines 230-231, line 266, line 307)

**Comment 5**. Line no. 221 after metric mentioning for precision and recall denominator "for precision, False Positives (FP)" and recall "False Negatives (FN):" Check the mentioned information and update accordingly.

***Response:*** Thank you for your valuable contribution. The mentioned information was adjusted (Line 220 – equations (3) and (4)).

**Comment** **6.** Equation no. 2: "gt" and "pr" full-length text abbreviation required.

***Response:*** We appreciate your suggestion. Full-length abbreviations of “gt” and “pr” were updated (Lines 221-222).

**Comment** **7.** Line no . 243 in Equation no. 7 right form was "F1 Score = 2 ×" Precision × Recall / Precision + Recall," but it mentioned "Precision - Recall."

***Response:*** We would like to send you our thankfulness for this comment. The equation was fixed (Line 245 – Equation (7)).

**Comment** **8.** Overall, all the equation numbering describes it before or after the numbering inside the paragraph with the reference number of the equation.

***Response:*** As comment 4, all the equation numbering was described after the numbering inside the paragraph with the reference number of the equation (Lines 208-210, line 219, lines 230-231, line 266).

**Comment** **9.** Line no. 264 in the equation number 9 description area mentions "N is the total number of models," which is not present in equation no. 9.

***Response:*** Thank you for reminding this problem. We have checked and fixed the equation (9) (Line 266).

**Comment** **10.** Figure 1 is required to be clear; the image is unclear and blurry.

***Response:*** Thanks for your contribution. The figures we updated have been checked and satisfied the request of the article. During the process of compressing all files into a PONE file, the image quality was automatically reduced. To obtain a clearer image, you can download it by clicking on "Click here to access/download;Figure;Fig1.tif”.

**Comment** **11.** Figure 2.b labelled image caption titles are not clear.

***Response:*** As comment 10, you can download the image for clearer information by clicking “Click here to access/download;Figure;Fig2_.tif”.

**Comment** **12.** Figure 5 caption titles—type it clearly

***Response:*** As comments 10 and 11, a better version of Figure 5 can be accessed by clicking “Click here to access/download;Figure;Fig5.tif”.
